# Expression of S100A Alarmins in Cord Blood Monocytes Is Highly Associated With Chorioamnionitis and Fetal Inflammation in Preterm Infants

**DOI:** 10.3389/fimmu.2020.01194

**Published:** 2020-06-16

**Authors:** Veronika Golubinskaya, Henri Puttonen, Ing-Marie Fyhr, Halfdan Rydbeck, Ann Hellström, Bo Jacobsson, Holger Nilsson, Carina Mallard, Karin Sävman

**Affiliations:** ^1^Department of Physiology, Institute of Neuroscience and Physiology, University of Gothenburg, Sahlgrenska Academy, Gothenburg, Sweden; ^2^Department of Pathology, Sahlgrenska University Hospital, Gothenburg, Sweden; ^3^Department of Clinical Neuroscience, Institute of Neuroscience and Physiology, University of Gothenburg, Sahlgrenska Academy, Gothenburg, Sweden; ^4^Department of Obstetrics and Gynecology, Institute of Clinical Science, University of Gothenburg, Sahlgrenska Academy, Gothenburg, Sweden; ^5^Department of Obstetrics and Gynecology, Sahlgrenska University Hospital, Gothenburg, Sweden; ^6^Department of Genetics and Bioinformatics, Domain of Health Data and Digitalization, Institute of Public Health, Oslo, Norway; ^7^Department of Pediatrics, Institute of Clinical Sciences, University of Gothenburg, Sahlgrenska Academy, Gothenburg, Sweden; ^8^Department of Neonatology, The Queen Silvia Children's Hospital, Sahlgrenska University Hospital, Gothenburg, Sweden

**Keywords:** alarmins, S100A8, S100A9, preterm birth, chorioamnionitis, fetal inflammation, monocytes, cord blood

## Abstract

**Background:** Preterm infants exposed to chorioamnionitis and with a fetal inflammatory response are at risk for neonatal morbidity and adverse outcome. Alarmins S100A8, S100A9, and S100A12 are expressed by myeloid cells and have been associated with inflammatory activation and monocyte modulation.

**Aim:** To study S100A alarmin expression in cord blood monocytes from term healthy and preterm infants and relate results to clinical findings, inflammatory biomarkers and alarmin protein levels, as well as pathways identified by differentially regulated monocyte genes.

**Methods:** Cord blood CD14+ monocytes were isolated from healthy term (*n* = 10) and preterm infants (<30 weeks gestational age, *n* = 33) by MACS technology. Monocyte RNA was sequenced and gene expression was analyzed by Principal Component Analysis and hierarchical clustering. Pathways were identified by Ingenuity Pathway Analysis. Inflammatory proteins were measured by Multiplex ELISA, and plasma S100A proteins by mass spectrometry. Histological chorioamnionitis (HCA) and fetal inflammatory response syndrome (FIRS) were diagnosed by placenta histological examination.

**Results:** S100A8, S100A9, and S100A12 gene expression was significantly increased and with a wider range in preterm vs. term infants. High S100A8 and S100A9 gene expression (*n* = 17) within the preterm group was strongly associated with spontaneous onset of delivery, HCA, FIRS and elevated inflammatory proteins in cord blood, while low expression (*n* = 16) was associated with impaired fetal growth and physician-initiated delivery. S100A8 and S100A9 protein levels were significantly lower in preterm vs. term infants, but within the preterm group high S100A gene expression, spontaneous onset of labor, HCA and FIRS were associated with elevated protein levels. One thousand nine hundred genes were differentially expressed in preterm infants with high vs. low S100A alarmin expression. Analysis of 124 genes differentially expressed in S100A high as well as FIRS and HCA groups identified 18 common pathways and S100A alarmins represented major hubs in network analyses.

**Conclusion:** High expression of S100A alarmins in cord blood monocytes identifies a distinct clinical risk group of preterm infants exposed to chorioamnionitis and with a fetal inflammatory response. Gene and pathway analyses suggest that high S100A alarmin expression also affects monocyte function. The connection with monocyte phenotype and inflammation-stimulated S100A expression in other cell types (e.g., neutrophils) warrants further investigation.

## Introduction

Preterm infants are at increased risk for severe neonatal morbidities as well as long term cognitive and motor impairment (1). The risk is inversely related to gestational age at birth (1), but there are also additional risk factors that may affect neonatal morbidities and long term outcome. One distinct clinical risk group are infants born to mothers with intrauterine infection and/or inflammation, commonly referred to as chorioamnionitis.

Chorioamnionitis is a major cause of preterm labor (PTL), preterm prelabor rupture of membranes (PPROM) and subsequent preterm delivery (2–4) and present in >50% of extremely preterm deliveries (5). Chorioamnionitis is commonly asymptomatic in the mother and spontaneous onset of labor or rupture of membranes are often the only clinical symptoms (5, 6). Reliable diagnosis is commonly obtained only after birth following histological examination of the placenta. Histological chorioamnionitis (HCA) is defined as a maternal inflammatory response in the placenta and may be associated with a fetal inflammatory response syndrome (FIRS) characterized by inflammation in fetal blood vessels and/or umbilical cord (funisitis) (5, 7). Preterm infants exposed to HCA and FIRS have an increased risk of early onset sepsis (8, 9) and may have elevated inflammatory parameters in cord blood as an additional sign of fetal inflammation (10, 11), but most infants have no clinical symptoms and there are no biochemical markers for FIRS in clinical use.

In spite of a lack of early symptoms, exposure to chorioamnionitis, and FIRS in particular, has been linked to an increased risk for severe neonatal morbidities (12–14), preterm brain injury (11, 15), and long term adverse outcomes (16, 17), suggesting that inflammatory mechanisms may contribute to injury. In addition to a possible risk of inflammation-induced injury, chorioamnionitis may also affect the immune response and the risk of severe infections in the preterm infant (18). It is thus of vital importance to characterize the fetal immune response associated with exposure to intrauterine infection/inflammation and to identify underlying mechanisms that may lead to inflammation-induced injury or affect the defense against infections. Recent studies suggest that monocyte phenotype is affected by the intrauterine environment and that alterations in monocyte function may change susceptibility to additional infectious or inflammatory insults (19, 20).

Alarmins are endogenous proteins/peptides that are released from cells in response to stress, immune activation, or cellular injury (21). Alarmins act as damage-associated molecular patterns (DAMPs) on pattern recognition receptors to initiate an immune-response (21). Proteins from the S100 family; S100A, S100A9, and S100A12 (also called calgranulin a, b, and c), are highly expressed in myeloid cells including neutrophils and monocytes (22). They make up a considerable part of cytoplasmic proteins in these cells (23), where they are important for phagocytosis and facilitate cell adhesion and migration (21). Neutrophil cells represent a larger circulating population than monocytes and also contain a larger percentage of S100A proteins and may therefore be a major source of proteins released under inflammatory conditions (23). When released following cellular injury or as a response to inflammatory stimuli, the S100A8 and S100A9 alarmins preferably form a heterodimer complex (calprotectin) and act as a DAMP by activating the RAGE and TLR4 receptors resulting in cytokine release and chemotaxis (21, 24). In the clinical setting, the S100A8/A9 dimer is released in response to local inflammatory processes into stool or plasma (25). In adults, it serves a preferable biomarker for certain inflammatory conditions such as inflammatory bowel disease and rheumatoid arthritis (25, 26).

S100A8/A9 has also been studied in the neonatal setting, mainly by studies of protein levels in association with various conditions. Protein levels of S100A8/A9 are increased in healthy term infants compared with adults and equal those found in adult patients during inflammation (19, 27), while preterm infants not exposed to chorioamnionitis have lower levels than term infants, but still significantly higher than in adults (19). S100A8/A9 proteins are also elevated in neonatal sepsis in preterm infants (28), while no elevation was seen in association with clinical intraamniotic infection (29). Recent studies also suggest that increased S100A8/A9 protein levels are responsible for specific characteristics of neonatal monocytes (19). In healthy term infants without signs of inflammation, S100A8/A9 modulates TLR responses resulting in a decreased risk for potentially tissue damaging hyperinflammation with preserved bacterial clearance. Low S100A8/A9 protein levels in cord blood are also associated with an increased risk of late onset sepsis in preterm infants (19). In addition, elevated levels of S100A9 and S100A12 proteins in amniotic fluid are associated with elevated inflammatory markers in cord blood and an increased risk for early onset sepsis (EOS) (30). To our knowledge, monocyte gene expression of S100A alarmins has not been studied in clinical inflammatory conditions or in the neonatal setting.

In this study, we investigate S100A8, S100A9, and S100A12 gene expression in cord blood monocytes from term healthy infants and preterm infants with exposure to chorioamnionitis or with a fetal inflammatory response. To characterize clinically relevant monocyte phenotypes, we relate our findings to clinical features, to inflammatory biomarkers and S100A proteins in cord blood and to potentially important pathways and networks identified by differentially regulated monocyte genes.

## Methods

### Study Population

The study was conducted at Sahlgrenska University Hospital, and at the Sahlgrenska Academy, University of Gothenburg, Sweden. The study was approved by the Regional Ethic's Committee (EPN Gbg 933-16, T350-18) at the Sahlgrenska University Hospital, Gothenburg, Sweden and children were enrolled following written informed parental consent. The preterm group consisted of 33 infants born at <30 weeks gestational age and the term group of 10 term infants born after normal vaginal delivery and without perinatal complications.

Data regarding maternal morbidity and pregnancy complications as well as data on deliveries and neonatal morbidities was obtained from medical charts. Pre-eclampsia was diagnosed in mothers with elevated blood pressure and significant proteinuria, and suspected clinical chorioamnionitis in mothers with fever >38.0°C and/or elevated CRP that received antibiotics on suspicion of intrauterine infection. Spontaneous onset of delivery was recorded when delivery was started by either spontaneous contractions (PTL) or rupture of membranes (PPROM) and physician-initiated delivery when infants were delivered by cesarean section without PTL or PPROM. Inflammatory markers were analyzed according to clinical routine within 1 h of birth and were considered elevated with CRP > 10 mg/L and/or Interleukin (IL)-6 >1,000 ng/L. Sepsis was diagnosed when clinical symptoms of infection were accompanied by positive blood cultures, except for *Staphylococcus epidermidis* where, in addition, a CRP > 20 mg/L was required for diagnosis. Additional severe neonatal morbidities were recorded, including intraventricular hemorrhage (IVH) grade 3–4, necrotizing enterocolitis (NEC) with clinical and radiological signs, patent ductus arteriosus (PDA) requiring medical or surgical treatment, and chronic lung disease (CLD) with oxygen need at 36 weeks gestational age.

### Placenta Histology

The diagnoses of HCA and FIRS were based on joint analyses by two trained perinatal pathologists. Tissue samples were obtained from umbilical cord (proximal and distal samples), roll of chorioamniotic membranes, umbilical cord insertion, and full-thickness samples of placenta. Histological examination of the placenta was performed following College of American Pathologists guidelines and findings were classified according to the ELGAN protocol as previously described (7). HCA was defined as a maternal inflammatory response with neutrophil infiltration of subchorionic space, chorionic plate, and amnion (Stages 1–3) while FIRS was defined as inflammation of the umbilical cord (funisitis) and/or neutrophilic infiltration of fetal stem vessels. Placentas from twins were examined and classified individually.

### Cord Blood Sampling and Storage

Cord blood was collected by gentle needle aspiration and transferred to EDTA tubes. After sampling, blood was stored vertically in closed tubes at +4°C without agitation until processing. Samples were stored for a median of 5.7 h (range 1.6–21.5 h). Storage time did not significantly affect gene expressions of S100A8, A9, or A12 (Spearman rank test, *p* > 0.05, correlation coefficients < 0.3).

### Blood Plasma Preparation and CD14+ Monocyte Isolation

Blood was mixed by repeated inversion of the tube and then spun at room temperature for 2 min at 2,000 × g. Plasma was separated to a new tube, spun for 5 min at 2,000 × g and the supernatant was aliquoted and saved frozen at −80°C until analysis.

Removed plasma was replaced by a corresponding volume of EasySep™ cell separation buffer (STEMCELL Technologies Inc., cat.#20144), and the cell pellet was carefully resuspended. For preparing the peripheral blood mononuclear cell (PBMC) fraction, the samples were diluted 1:2 in EasySep™ cell separation buffer and spun with brakes off in Lymphoprep™ density gradient medium (STEMCELL Technologies Inc., cat.#07801) at 1,200 × g for 20 min at room temperature.

All further steps of the monocyte isolation protocol were conducted at +4°C. CD14+ monocytes were purified from PBMC fraction with the help of magnetic cell separation (MACS) technology according to the manufacturer protocol (CD14 MicroBeads, human, cat.#130-050-201; autoMACS running buffer, cat.#130-091-221; MiniMACS Separation columns, type MS, cat.#130-042-201; Miltenyi Biotec). The last washing step was made with D-PBS without calcium and magnesium (STEMCELL Technologies Inc., cat.#37350). The CD14+ monocyte cell pellets were then stored at −80°C for later RNA extraction.

Prior to the last washing step the purified monocytes were counted and number of dead cells was determined based on positive TrypanBlue staining. Cell samples were also placed on +-charged glasses, quickly dried, fixed with ice-cold acetone-methanol and stored frozen at −20°C for further analysis of the monocyte fraction purity. Cells on the slides were stained with anti-CD14+ antibodies (1:200, HPA001887, Atlas Antibodies AB), and the number of positive cells, as well as the morphology of the cells and the cell nuclei were evaluated. Purity of the CD14+ monocyte fraction was >96% and cell viability on average 93%.

### RNA Sequencing

Monocyte cell pellets were homogenized in QIAzol Lysis Reagent (Qiagen, cat.# 79306) and then total RNA was purified by miRNeasy Micro Kit (Qiagen, cat.# 217084) according to the manufacturer's protocol. RNA quantity and purity were analyzed by NanoDrop (Thermo Fisher Scientific), and RNA integrity was determined by Experion™ automated electrophoresis system (BioRad). Samples that proceeded to RNA sequencing had a mean (SD) RQI of 9.2 (1.0). RNA sequencing experiments and basic data analysis were conducted at QIAGEN Genomic Services, as described below.

### Library Preparation and Next Generation Sequencing

The library preparation was done using Illumina TruSeq Stranded Total RNA Library Prep Kit with rRNA depletion (Illumina Inc.). A total of 100 ng RNA was used. Sequencing was performed on a NextSeq500 instrument, with an average number of reads 60 million paired-end reads/per sample and number of sequencing cycles (read length) 2 × 75 nt. Sequencing was performed according the manufacturer's instructions (Illumina Inc.). The NGS data analysis pipeline was based on the Tuxedo software package. Briefly, abundant sequences were filtered after aligning the raw data with Bowtie2. Tophat was used to perform alignment to the reference genome (GRCh37, annotation from ENSEMBL_75). Cufflinks was used to assemble transcripts, Cuffmerge to merge transcripts and Cuffquant to quantify expression levels. Counts were converted to fpkm (fragments per kilobase million) values and the data set was filtered.

After preparing library, all samples passed the internal quality check (Qiagen, Genomic Services). Following sequencing, intensity correction and base calling (into BCL files), and FASTQ files were generated using the appropriate bcl2fastq software (Illumina Inc.). Average read *Q*-score was above 30 (high quality data). On average 33.0 million reads were obtained for each sample and the average genome mapping rate was 90.5%. All samples in the study had similar call rates (similar numbers of genes identified) and were considered to be comparable. In the analysis the fpkm values are normalized with median of the geometric mean (31).

Further analyses included only the transcripts where, in all groups, 10% or more of individual reads were at least 2 fpkm. Unmapped transcripts, clone-based genes, ribosome-related RNA transcripts, small nuclear and nucleolar RNA-related transcripts, Y-RNA and non-unique transcripts were also eliminated. Zero fpkm values for the individual reads were replaced by 1 fpkm, and then all fpkm values were transformed to log2 fpkm values for further analysis.

To validate RNA sequencing results, qPCR analysis was conducted at QIAGEN Genomic Services on 4 samples each from high and low S100A preterm groups as identified by RNAseq. Samples were analyzed using a PCR array panel (Human aging panel, Cat.# PAHS-178Z, product # 33023) which included S100A8 and S100A9, as well as 5 additional genes from the top 500 differentially expressed (DE) genes between S100 high and S100 low groups (ANXA3, FCER1G, FCGR1A, LMNB1, WRN). Five housekeeping genes (ACTB, B2M, GAPDH, HPRT1, RPLP0) were also included. The average of Ct values for the housekeeping genes were calculated, and for further analysis delta Ct for each gene was used (dCt = average Ct for 5 housekeeping genes – Ct for the gene of interest). In the sequencing data analysis log2(fpkm) values were used.

### Measurements of Inflammatory Proteins in Plasma

A comprehensive screening of plasma cytokines, chemokines, and growth factors was performed by Bio-Plex ELISA (Bio-Rad Laboratories, Inc., Bio-Plex Pro™ Human Cytokine Screening Panel, 48-Plex #12007283, Bio-Plex Pro™ Human Inflammation Panel 1, 37-Plex #171AL001M) according to the manufacturer's instructions. Samples were diluted according to the manufacturer protocol (1:4 in sample diluent). Low “out-of-range” values were replaced by 1/8 of the detection limit value for the protein measured. No high “out-of-range” values were registered. For the proteins that were included in both of the Bio-Plex panels, reads from only one assay were included, based on best fit of the measurements to the standard curve.

### Quantitative Analysis of Plasma S100A8 and S100A9 Proteins by Mass Spectrometry

Each plasma sample (0.6 μl) was reduced in 100 mM DTT with 2% SDS and 50 mM TEAB, and processed by the FASP method (32) including alkylation with 10 mM MMTS, and digestion using trypsin (MS Grade, Thermo Fisher Scientific). Peptides were labeled with TMT 11-plex (Thermo Fisher Scientific), pooled per TMT set, and subjected to basic reverse-phase fractionation. The 40 collected fractions were pooled into 20, and analyzed on an Easy nanoLC 1200 liquid chromatography system, coupled to an Orbitrap Fusion Lumos Tribrid instrument (Thermo Fisher Scientific). Peptides were trapped on an Acclaim Pepmap 100 C18 trap column (100 μm × 2 cm, particle size 5 μm, Thermo Fischer Scientific) and separated on an in-house packed analytical column (75 μm × 300 mm, particle size 3 μm, Reprosil-Pur C18, Dr. Maisch) using a gradient from 5 to 100% acetonitrile in 0.2% Formic Acid. The nLC MS analysis was performed in a data-dependent multinotch mode using an m/z of 400–1,400 and a dynamic exclusion of 45 s.

Identification and relative quantification was performed using Proteome Discoverer v. 2.2 (Thermo Fisher Scientific). Mascot v. 2.5.1 (Matrix Science, London, UK) was used to match to the H. sapiens database (SwissProt, September 2019) with MS peptide tolerance of 5 ppm and fragment ion tolerance of 0.6 Da. Miscleavages were set on 0, methionine oxidation was set as variable modification; cysteine methylthiolation; and TMT-modification were set as fixed. Percolator was used for PSM validation with an FDR threshold of 1%. Normalization of all TMT reporter intensities for each sample on the total peptide amount was performed. Only unique identified peptides were used for relative quantification, and ratios were calculated by dividing the samples with the reference sample.

### Gene Co-expression Network Analysis

Only samples from preterm infants were analyzed. For network analysis the expression values of genes left after filtering (10,533 genes) were processed with the R package WGCNA (33) following a pipeline adapted from tutorial I, available from the website (https://horvath.genetics.ucla.edu/html/CoexpressionNetwork/Rpackages/WGCNA/). The WGCNA function for network and module construction, blockwiseModules, uses the soft threshold (power), the minimum module size and mergeCutHeight parameters when calculating the network and the modules, which in turn will affect the GO analysis. We used the settings in the tutorial, except for using minimum module size of 100. For each module, representative eigengenes were defined as the first principal component of gene expression in a given module. The correlation between eigengenes (Pearson correlation) and S100A high or low expression, as well as clinical conditions (FIRS, HCA, PTL/PPROM, elevated CRP/IL-6) was determined. The Gene Ontology Enrichment analysis was performed with the WGCNA function GO enrichment Analysis. Networks were exported for filtering and visualization in the software VisAnt. For selecting hub genes, we used code adapted from Tutorial III, section 7. Connectivity for each gene was calculated with the WGCNA function intramodular Connectivity. This function sums all adjacency entries to other genes, both within the module for intramodular connectivity and to all analyzed genes for total connectivity.

Hub-genes were selected as the top 5% genes with the highest within-module connectivity in order to capture genes with the highest connectivity according to the connectivity distribution (34). The adapted code is available at https://github.com/halryd/~high_S100A_hub_genes.

### Data Analysis

For creating lists of differentially expressed genes and inflammatory proteins for principal component analysis (PCA), for hierarchical clustering, and for visual representation in the form of heat maps, the commercial software Qlucore Omics Explorer v3.4 (Qlucore AB, Sweden) was used. Pathway analysis was performed by commercial software Ingenuity Pathway Analysis (IPA) (Qiagen Bioinformatics). Network co-expression analysis is described in detail above.

Clinical data, single genes and proteins were compared using unpaired *t*-tests or Fisher's exact test, following either normality tests or log transformation (GraphPad Prism Software). A *p* < 0.05 was considered statistically significant.

Statistical comparisons of groups of parameters (genes and inflammatory proteins) between study groups were made using Qlucore software (multiple *t*-tests with Benajmini-Hochberg multiple correction procedure). A *q* < 0.05 was considered statistically significant.

Correlations were calculated as either Spearman's rank correlation coefficient, or Pearson's correlation coefficient as appropriate, and correction for confounding factors was performed by linear regression.

## Results

### Gene Expression in Cord Blood Monocytes and Plasma Inflammatory Protein Profiles Differ Between Preterm and Term Infants

**Figure 1:**
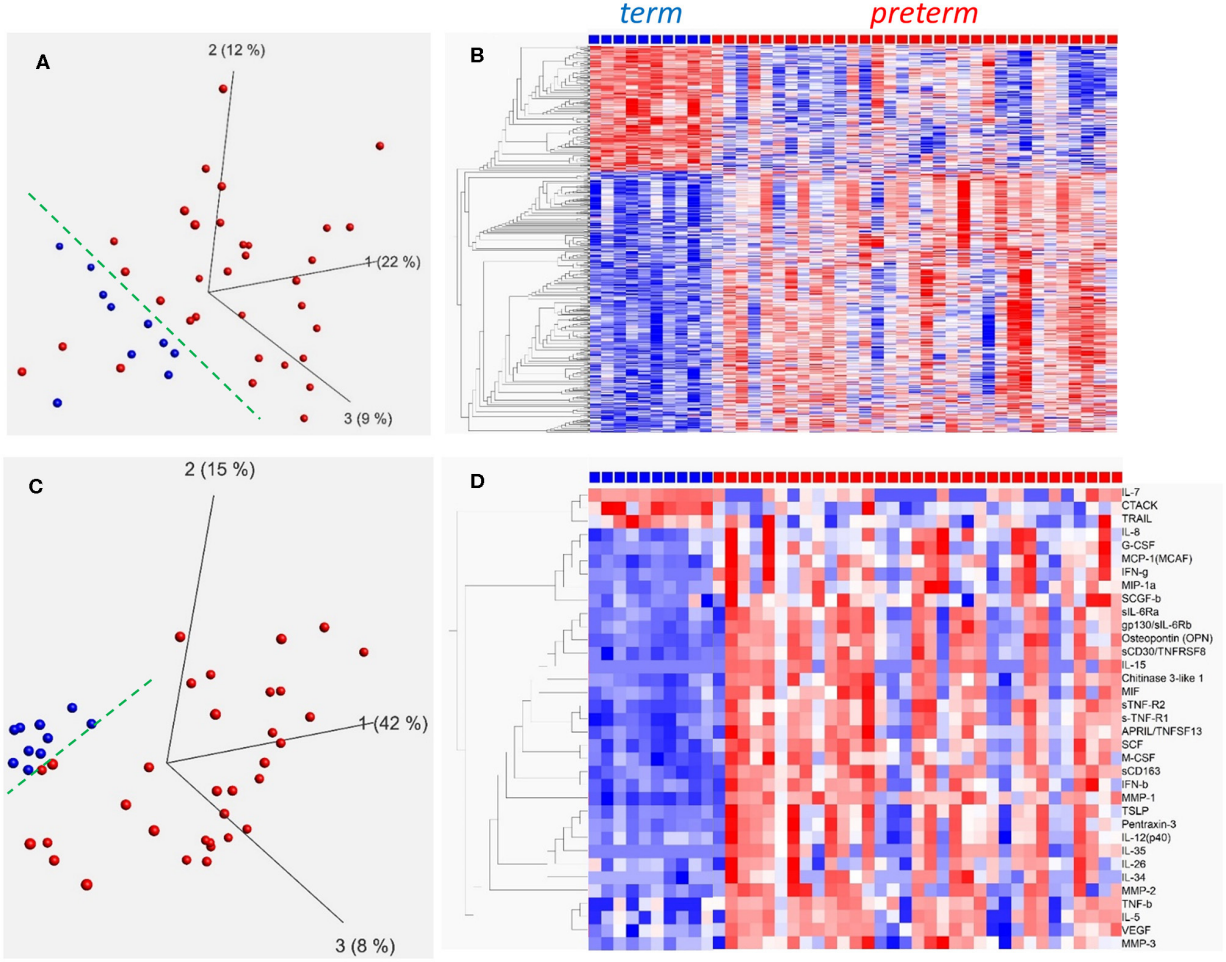
Gene expression in CD14+ cord blood monocytes **(A,B)** and plasma inflammatory protein profiles **(C,D)** in term and preterm infants presented by PCA analysis **(A,C)** and hierarchical clustering **(B,D)**. Principal component analysis (PCA) plots for monocyte gene expression **(A)** and plasma inflammatory proteins **(C)** indicate that term infants (blue, *n* = 10) are better grouped than preterm infants (red, *n* = 33). Heat maps with hierarchical clustering present top 500 differentially expressed genes (*q* < 0.008, Qlucore software; multiple *t*-tests with Benajmini-Hochberg multiple correction procedure) in monocytes **(B)** and all significantly different (*q* ≤ 0.049) inflammatory proteins in plasma **(D)**. Heatmaps and hierarchical clustering are based on fpkm (fragments per kilobase million) values where each value is normalized to mean = 0 and variance = 1. Red color denotes up-regulation and blue down-regulation within a range of −2 to +2.

In a first set of experiments we investigated cord blood monocyte gene expression in preterm (*n* = 33) and term (*n* = 10) infants. Background data on infants included is shown in Table 1. In summary, 1,924 genes were differentially expressed between the preterm group and the healthy term group. PCA analyses of all genes (Figure 1A) and heat maps based on hierarchical clustering of top 500 differentially expressed (DE) genes (Figure 1B, *q* < 0.008) show a homogenous pattern of expression within the term group. The preterm group is distinctly different from the term group, and demonstrates a significantly larger variability with clear differences in gene expression between individual preterm infants.

**Table 1:** Background data on preterm and term infants.

	**Preterm infants (*n* = 33)**	**Term infants (*n* = 10)**
Gestational age (days), mean (SD)	185 (14)	284 (7)
Gestational age (weeks + days), median (range)	26 + 3 (23 + 0 – 29 + 5)	40 + 5 (38 + 2 – 41 + 4)
Birth weight (g), mean (SD)	920 (307)	3,620 (520)
Boys (%)	23/33 (69.7)	7/10 (70)

To investigate whether differences in inflammatory activation could help to explain differences in gene expression between preterm and term infants we performed gene pathway analyses. An overview of the 20 most affected pathways based on DE genes are listed in Table 2. In summary, more than half of the identified pathways were clearly related to inflammation and immune regulation, including several pathways associated with monocyte maturation and, in particular, monocyte recruitment and extravasation.

**Table 2:** Top 20 canonical IPA pathways differentially regulated in cord blood monocytes from preterm and term infants.

**Ingenuity canonical pathways**	**Signaling pathway categories (IPA)**	**–log** **(*p*-value)**	**Relation to inflammation and/or monocyte function (select references)**
Regulation of Actin-based Motility by Rho	Neurotransmitters and Other Nervous System Signaling	5.72	Rho is a family of GTPases, important in innate and adaptive immunity (35)
Thrombin Signaling	Cardiovascular Signaling	5.54	Involved in monocyte regulation of systemic coagulation in (36)
RhoGDI Signaling	Intracellular and Second Messenger Signaling	4.98	Involved in Rho-regulation; important for innate and adaptive immunity (35, 37)
Integrin Signaling	Cell Cycle Regulation; Cellular Growth, Proliferation and Development; Intracellular and Second Messenger Signaling	4.75	Critical in monocyte trafficking and vessel wall adhesion (38)
Actin Cytoskeleton Signaling	Cell Cycle Regulation; Cellular Growth, Proliferation and Development; Intracellular and Second Messenger Signaling	4.63	Regulates locomotion, phagocytosis, and cell shape in leukocytes including monocytes (39)
Relaxin Signaling	Growth Factor Signaling; Organismal Growth and Development	4.5	An insulin-like peptide with properties important for recruitment of peripheral blood mononuclear cells to sites of inflammation (40)
Cellular Effects of Sildenafil (Viagra)	Cardiovascular Signaling; Disease-Specific Pathways	4.34	N/A
Ephrin B Signaling	Neurotransmitters and Other Nervous System Signaling; Organismal Growth and Development	4.15	Ephrin proteins are involved in inflammation in vascular endothelium (41) including in monocytes (42)
Signaling by Rho Family GTPases	Intracellular and Second Messenger Signaling	4.11	Rho is a family of GTPases, important in innate and adaptive immunity (35)
Molecular Mechanisms of Cancer	Cancer; Disease-Specific Pathways	4.1	N/A
Cardiac β-adrenergic Signaling	Cardiovascular Signaling	4.07	N/A
Protein Kinase A Signaling	Intracellular and Second Messenger Signaling	4	N/A
Synaptogenesis Signaling Pathway	Neurotransmitters and Other Nervous System Signaling; Organismal Growth and Development	3.91	N/A
Leukocyte Extravasation Signaling	Cellular Immune Response	3.9	Monocytes immune surveillance and trafficking across vasculature (42)
Germ Cell-Sertoli Cell Junction Signaling	Cellular Growth, Proliferation and Development	3.89	N/A
IL-8 Signaling	Cellular Immune Response; Cytokine Signaling	3.88	Commonly found in early-onset neonatal sepsis (43)
Breast Cancer Regulation by Stathmin1	Cancer; Disease-Specific Pathways	3.86	N/A
Epithelial Adherens Junction Signaling	Cellular Growth, Proliferation and Development	3.73	N/A
Androgen Signaling	Nuclear Receptor Signaling	3.52	N/A
CXCR4 Signaling	Cellular Immune Response; Cytokine Signaling	3.4	Involved in maturation and replenishment of monocytes (44)

*Analysis is based on 500 genes differentially expressed between term and preterm groups and analyzed by Ingenuity Pathway Analysis (IPA) (Qiagen Bioinformatics)*.

In addition, inflammatory plasma proteins were analyzed by Bio-Plex Elisa, and concentrations differed between preterm and term groups for 36 out of 77 measured proteins (Figure 1D, *q* < 0.049). Similar to gene expression, term infants exhibited a homogenous protein profile while the preterm group included infants with varying protein patterns thus suggesting differences in inflammatory phenotypes within the preterm group as demonstrated by PCA analysis (Figure 1C) and heat map (Figure 1D). Detailed data on inflammatory protein analyses is found in Supplementary Tables 1, 2.

### Preterm Infants Have a Different Pattern of S100A Alarmin Expression in Cord Blood Monocytes Compared With Term Infants

**Figure 2:**
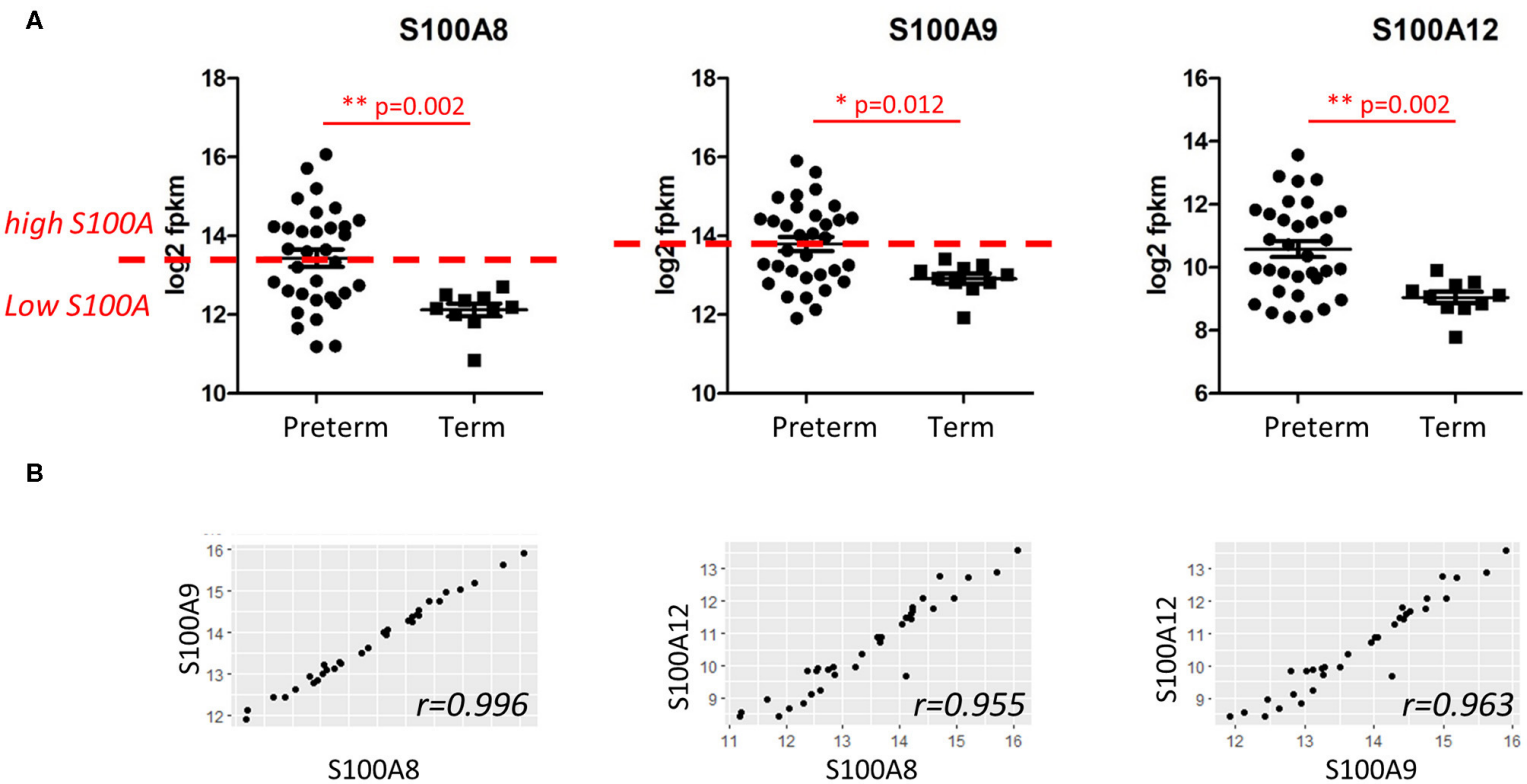
Expression of S100A alarmin genes in CD14+ cord blood monocytes from preterm and term infants. **(A)** Expression of S100A8, S100A9, and S100A12 alarmins was significantly higher and with a wider range in preterm (*n* = 33) compared to term infants (*n* = 10). Data presented as mean ± SEM and compared with unpaired *t*-test for log2-transformed fpkm (fragments per kilobase million) values, *p*-values in figure. Red line indicate division of preterm infants into a S100A high group (S100A8 and S100A9 at or above median, *n* = 17) and a S100A low group (S100A8 and S100A9 below median, *n* = 16). **(B)** Gene expressions of the different S100A alarmins were strongly correlated with each other, Pearson's correlation coefficients > 0.95.

Since S100A alarmins are associated with inflammatory activation and modulate monocyte function in the neonatal period (19), gene expression for alarmins S100A8, S100A9, and S100A12 in cord blood monocytes was analyzed separately and compared between preterm and term infants. Expression was significantly higher and had a wider range in preterm infants for all three genes (Figure 2A).

To further investigate the association of alarmin expression and inflammatory phenotypes in the preterm group, we divided the infants into two groups using the median log2 fpkm expression for the different genes (Figure 2A, red line). In our experiments, gene expressions of the different S100A alarmins were strongly correlated with each other with correlation coefficients > 0.95 (Figure 2B). As S100A8 and S100A9 proteins commonly form biologically active heterodimers and gene expression of S100A8 and S100A9 identified exactly the same infants with high (*n* = 17, at or above median) and low (*n* = 16, under median) gene expression, these groups were used for further analyses and referred to as “S100A high” and “S100A low,” respectively. All term infants had alarmin gene expression defined as low by this division.

qPCR analysis was performed for S100A8, S100A9 and 5 of the top 500 DE genes between S100A high and S100A low groups to confirm RNA sequencing results. In summary, gene expression by qPCR was strongly correlated with RNA sequencing data (Table 3) and differences between groups remained significant for all genes examined (Supplementary Table 3).

**Table 3:** Correlation between gene expression analyzed by RNA sequencing or qPCR for S100A8, S100A9 and 5 genes differentially expressed between preterm infants with high or low S100A expression in cord blood monocytes.

**Gene**	***R***	**Slope**	***p*-value**
*S100A8*	0.97	1.23	0.0001
*S100A9*	0.95	1.20	0.0003
*ANXA3*	1.00	1.09	<0.0001
*FCER1G*	0.92	1.71	0.0013
*FCGR1A*	0.94	1.43	0.0005
*LMNB1*	0.99	1.24	<0.0001
*WRN*	0.77	0.54	0.0259

*N = 8, r, Pearson's correlation coefficient; slope, linear regression coefficient; P, P-values for r*.

### High Expression of S100A Alarmins in Cord Blood Monocytes From Preterm Infants Is Associated With Clinical Features Associated With Chorioamnionitis and Fetal Inflammation

Clinical background data on preterm infants divided by high or low alarmin S100A gene expression is presented in Table 4. High expression of S100A alarmins in cord blood monocytes was associated with several conditions indicative of chorioamnionitis and fetal inflammation. Infants with high expression of S100A alarmins were born at significantly lower gestational ages and all mothers had spontaneous onset of delivery with either PTL or PPROM and with a significantly higher proportion of HCA and FIRS. Elevated CRP and/or IL-6 in clinical routine samples from cord blood were found in the high S100A group only, while the only significant difference regarding neonatal morbidities was a higher percentage in need of treatment for patent ductus arteriosus in the high S100A group. Low S100A expression was associated with a different clinical risk profile, with a significantly higher number of small for gestational age (SGA) infants, with lower mean weight for gestational age and a higher proportion of physician-initiated deliveries. Placenta data was missing in six preterm infants, four in the S1000A low group and two in the S100A high group. Only two out of 11 infants with FIRS were found in the low S100A group. Interestingly, one was a twin with a sibling in the S100A high group. Both twins filled criteria for FIRS and exposure to HCA, but differed in degree of neutrophil infiltration.

**Table 4:** Clinical data on preterm infants divided by high or low gene expression of S100A alarmins in cord blood monocytes.

	**High expression of S100A (*n* = 17)**	**Low expression of S100A (*n* = 16)**	***p*-value**
Gestational age (days), mean (SD)	177 (11)	193 (11)	<0.001
Gestational age (weeks + days), median (range)	25 + 6 (23 + 0 – 27 + 5)	27 + 6 (24 + 6 – 29 + 5)	
Birth weight (g), mean (SD)	825 (220)	1,020 (360)	ns
Boys	14/17 (82.4%)	9/16 (56.2%)	ns
Standard deviation score (SDS) for weight, mean (SD)	−0.047 (0.73)	−1.49 (1.63)	0.02
Small for Gestational Age (SGA) < −2 SD for weight	0/17 (0%)	4/16 (25%)	<0.05
Twin infants	4/17 (23.5%)	6/16 (37.5%)	ns
Preeclampsia	0/17 (0%)	2/16 (12.5%)	ns
Suspected clinical chorioamnionitis	6/17 (35.3%)	5/16 (41.2%)	ns
Antenatal steroids	17/17 (100%)	16/16 (100%)	ns
Preterm Labor (PTL)	7/17 (41.2%)	4/16 (25%)	ns
Preterm Prelabor Rupture of Membranes (PPROM)	10/17 (58.8%)	5/16 (31.2%)	ns
Spontaneous onset of delivery (PTL/PPROM)	17/17 (100%)	9/16 (56.2%)	0.03
Physician-initiated delivery	0/17 (0%)	7/16 (43.8%)	0.03
Delivered by cesarean section	8/17 (47.1%)	12/16 (75%)	ns
Histological chorioamnionitis (HCA)	12/15 (80%)	2/12 (16.7%)	0.002
Fetal Inflammatory Response Syndrome (FIRS)	9/15 (60%)	2/12 (16.7%)	<0.05
Infant elevated CRP and/or IL-6 at birth	5/17 (29.4%)	0/16 (0%)	<0.05
Early onset sepsis (<3 d of age)	1/17 (5.8%)	0/16 (0%)	ns
Late onset sepsis (>3 d of age)	0/14 (0%)	2/14 (14.3%)	ns
Death	1/17 (5.8%)	2/16 (12.5%)	ns
Intraventricular hemorrhage (IVH) grade 3–4	2/17 (11.8%)	3/16 (18.8%)	ns
Necrotizing Enterocolitis (NEC)	2/17 (11.8%)	1/16 (6.2%)	ns
Patent ductus arteriosus (PDA)	11/17 (76.5%)	3/14 (21.4%)	0.03
Chronic Lung Disease (CLD)	8/15 (53.3%)	5/14 (35.7%)	ns

*Following normality tests, groups were compared using unpaired t-test or Fisher's Exact Test. Placenta data was not available in all infants and PDA and CLD could only be diagnosed in infants that survived until examination*.

**Figure 3:**
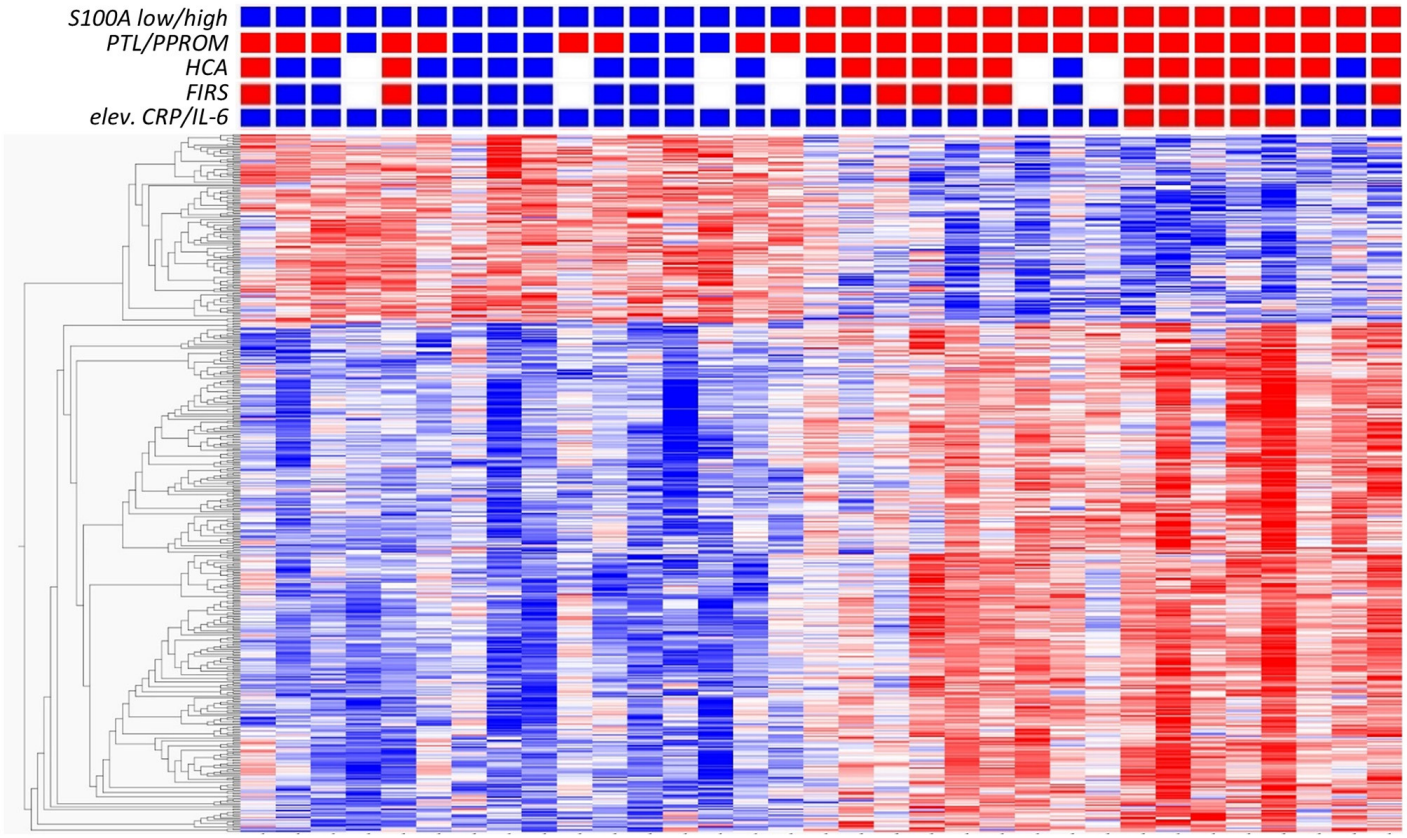
Analysis of gene expression in CD14+ cord blood monocytes in preterm infants based on expression levels of S100A alarmins and in relation to clinical features associated with chorioamnionitis and fetal inflammation. Heat map with hierarchical clustering of top 500 DE genes between high S100A (red squares, *n* = 17) and low S100A (blue squares, *n* = 16) groups (*q* < 0.0021, Qlucore software, multiple *t*-tests with Benajmini-Hochberg multiple correction procedure). Red squares also indicate presence of preterm labor (PTL)/preterm prelabor rupture of membranes (PPROM); exposure to histological chorioamnionitis (HCA), histological fetal inflammatory response syndrome (FIRS) and elevated CRP/IL-6 in cord blood plasma while blue squares indicate the absence of these conditions. White squares indicate missing data. Heatmaps and hierarchical clustering are based on fpkm (fragments per kilobase million) values where each value is normalized to mean = 0 and variance = 1. Red color denotes up-regulation and blue down-regulation within a range of −2 to +2.

To further investigate the association between S100A gene expression and risk groups, we identified a set of clinical conditions associated with chorioamnionitis and fetal inflammation, namely PTL/PPROM (spontaneous onset of delivery), HCA, FIRS and elevated CRP/IL-6. We then compared total gene expression between high (*n* = 17) and low (*n* = 16) S100A groups with respect to these clinical conditions. Figure 3 presents a heat map based on the 500 top DE genes (*q* < 0.002) with added clinical characteristics of the individual infants, showing a clustering of inflammatory features within the S100A high expression group.

**Figure 4:**
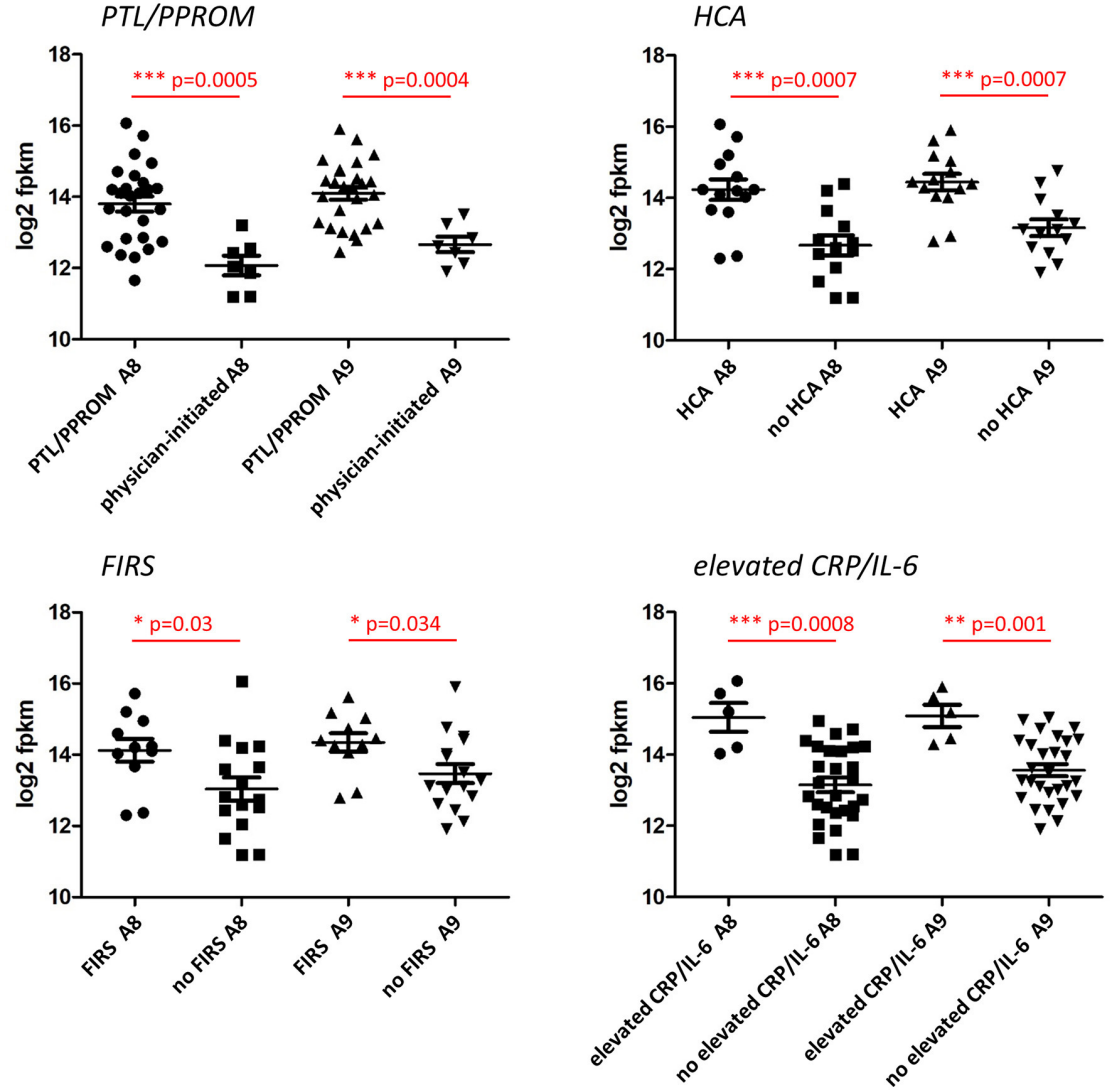
Expression of S100A alarmin genes in CD14+ cord blood monocytes in relation to clinical features associated with chorioamnionitis and fetal inflammation within the preterm group. Monocyte gene expression levels of S100A8 and S100A9 were significantly elevated in association with preterm labor (PTL)/preterm prelabor rupture of membranes (PPROM), exposure to histological chorioamnionitis (HCA), histological fetal inflammatory response syndrome (FIRS) as well as elevated CRP/IL-6 in cord blood plasma (individual samples demonstrated in figure). Data presented as mean ± SEM and compared with unpaired *t*-test for log2-transformed fpkm (fragments per kilobase million) values, *p*-values in figure.

In additional analyses of the association between S100A8 and S100A9 monocyte gene expression and clinical characteristics, we found that gene expression of both genes was significantly elevated in association with PTL/PPROM, HCA, FIRS, and elevated IL-6/CRP (Figure 4). A similar pattern with significant differences for all clinical groups was seen for S100A12 (data not shown).

Infants in the high S100A group were born at significantly lower gestational age than those in the low S100A group (Table 4). To exclude that gestational age in itself could explain differences in S100A expression, a linear regression was performed to adjust for gestational age as a confounding factor. In summary, regression analyses showed that gestational age alone could not explain differences in S100A gene expression between groups (Supplementary Figure 1).

### High Expression of S100A Alarmins in Cord Blood Monocytes Is Associated With Elevated Inflammatory Proteins in Cord Blood Plasma

**Figure 5:**
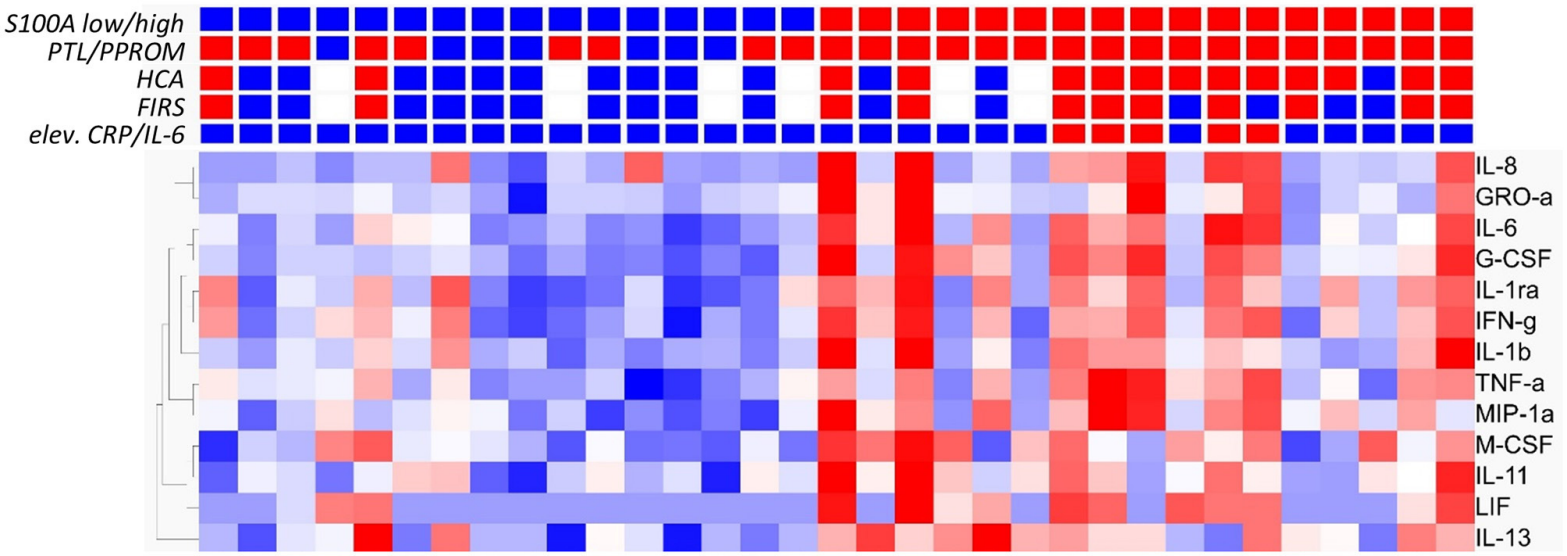
Analysis of inflammatory proteins in cord blood plasma from preterm infants based on expression levels of S100A alarmins and in relation to clinical features associated with chorioamnionitis and fetal inflammation. Heat map with hierarchical clustering demonstrates significant elevation of 13/77 inflammatory proteins analyzed by Multiplex ELISA in S100A high (red squares, *n* = 17) and S100A low (blue squares, *n* = 16) groups (*q* ≤ 0,049, multiple *t*-tests with Benajmini-Hochberg multiple correction procedure). Red squares also indicate presence of preterm labor (PTL)/preterm prelabor rupture of membranes (PPROM); exposure to histological chorioamnionitis (HCA), histological fetal inflammatory response syndrome (FIRS) and elevated CRP/IL-6 in cord blood plasma while blue squares indicate the absence of these conditions. White squares indicate missing data. Heatmaps and hierarchical clustering are based on fpkm fragments per kilobase million) values where each value is normalized to mean = 0 and variance = 1. Red color denotes up-regulation and blue down-regulation within a range of −2 to +2.

To determine if high expression of S100A alarmins was associated also with biochemical markers for inflammation, inflammatory proteins in cord blood plasma were analyzed in relation to alarmin expression. Thirteen out of 77 inflammatory proteins were increased in the high alarmin group as demonstrated in Figure 5, *q* < 0.049. The heat map also demonstrates a clear relation between an inflammatory protein pattern and clinical conditions associated with chorioamnionitis and fetal inflammation. In spite of this correlation, the differences between clinical groups regarding elevated inflammatory proteins were less pronounced than for groups with different S100A expression. Eleven out of 77 proteins were elevated in infants with FIRS (*n* = 11) vs. no FIRS (*n* = 16); 10/77 proteins in infants exposed to HCA (*n* = 14) vs. no HCA (*n* = 13); 7/77 in infants with elevated CRP/IL-6 (*n* = 5) vs. infants without elevated markers (*n* = 28) and no significantly elevated proteins were found in cord blood from infants born after spontaneous onset of delivery (PTL/PPROM, *n* = 26) compared with physician-initiated delivery (*n* = 7). Detailed data is found in Supplementary Table 1.

### Alarmin Proteins Are Elevated in Cord Blood Plasma From Preterm Infants With High Expression of S100A8 and S100A9 Genes in Monocytes and in Association With Spontaneous Onset of Labor, HCA and FIRS

**Figure 6:**
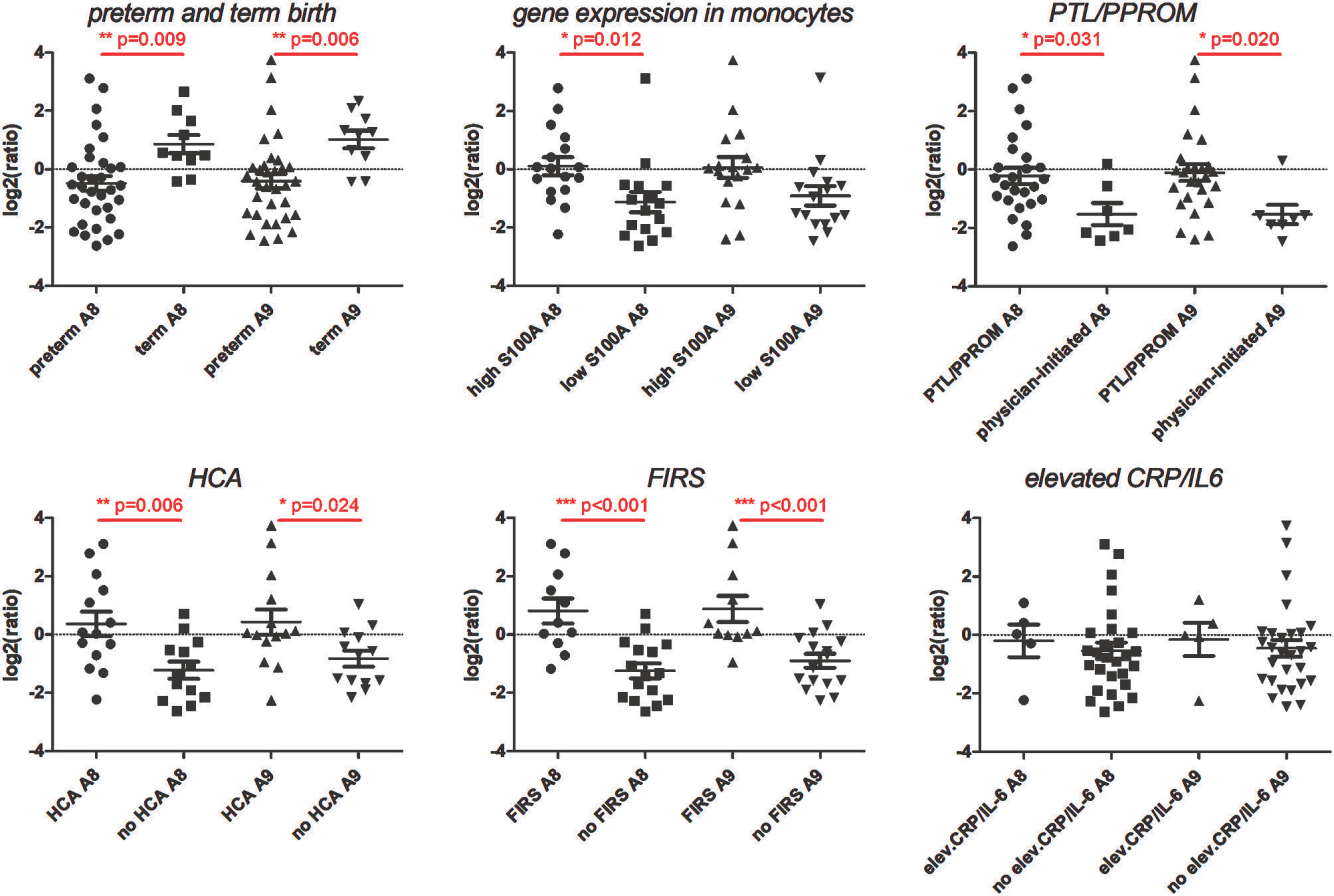
S100A8 and S100A9 proteins in cord blood plasma in preterm and term newborns and in relation to S100A gene expression and clinical features associated with chorioamnionitis and fetal inflammation within the preterm group. Plasma protein levels of S1000A8 and S100A9 were analyzed by mass-spectrometry and relative concentrations were compared between groups. The first panel shows that term infants (*n* = 10) had significantly higher levels than preterm infants (*n* = 33). The remaining panels all refer to differences in plasma protein levels within the preterm group. Elevated plasma protein levels were seen in infants with high (*n* = 17) compared with low (*n* = 16) S100A8 and S100A9 gene expression. Preterm labor (PTL)/preterm prelabor rupture of membranes (PPROM), histological chorioamnionitis (HCA), and histological fetal inflammatory response syndrome (FIRS) were also associated with increased protein levels, while exposure to elevated CRP/IL-6 in cord blood plasma was not (individual samples demonstrated in figure). Data presented as mean ± SEM and compared with unpaired *t*-test for log2-transformed relative concentrations of S100A8 and S100A9 proteins, *p*-values in figure.

To evaluate alarmin protein levels in preterm vs. term infants and in relation to clinical characteristics in the preterm group, proteins S100A8 and S100A9 were analyzed in cord blood plasma from term infants (*n* = 10) and in all preterm infants where monocyte gene expression was analyzed and with either high (*n* = 17) or low (*n* = 16) S100A8 and S100A9 gene expression. Protein levels were significantly higher in the term vs. the preterm group. Within the preterm group, spontaneous onset of labor (PTL/PPROM), HCA and FIRS were associated with elevated S100A8 and S100A9 plasma protein levels. High S100A monocyte gene expression was associated with elevated plasma levels of protein S100A8, while the elevation of protein S100A9 was borderline significant (*p* = 0.056) (Figure 6).

### Multiple Differentially Expressed Genes and Several Pathways Are Common to Preterm Infants With High Expression of S100A Alarmins and Clinical Features Associated With Chorioamnionitis and Fetal Inflammation

**Figure 7:**
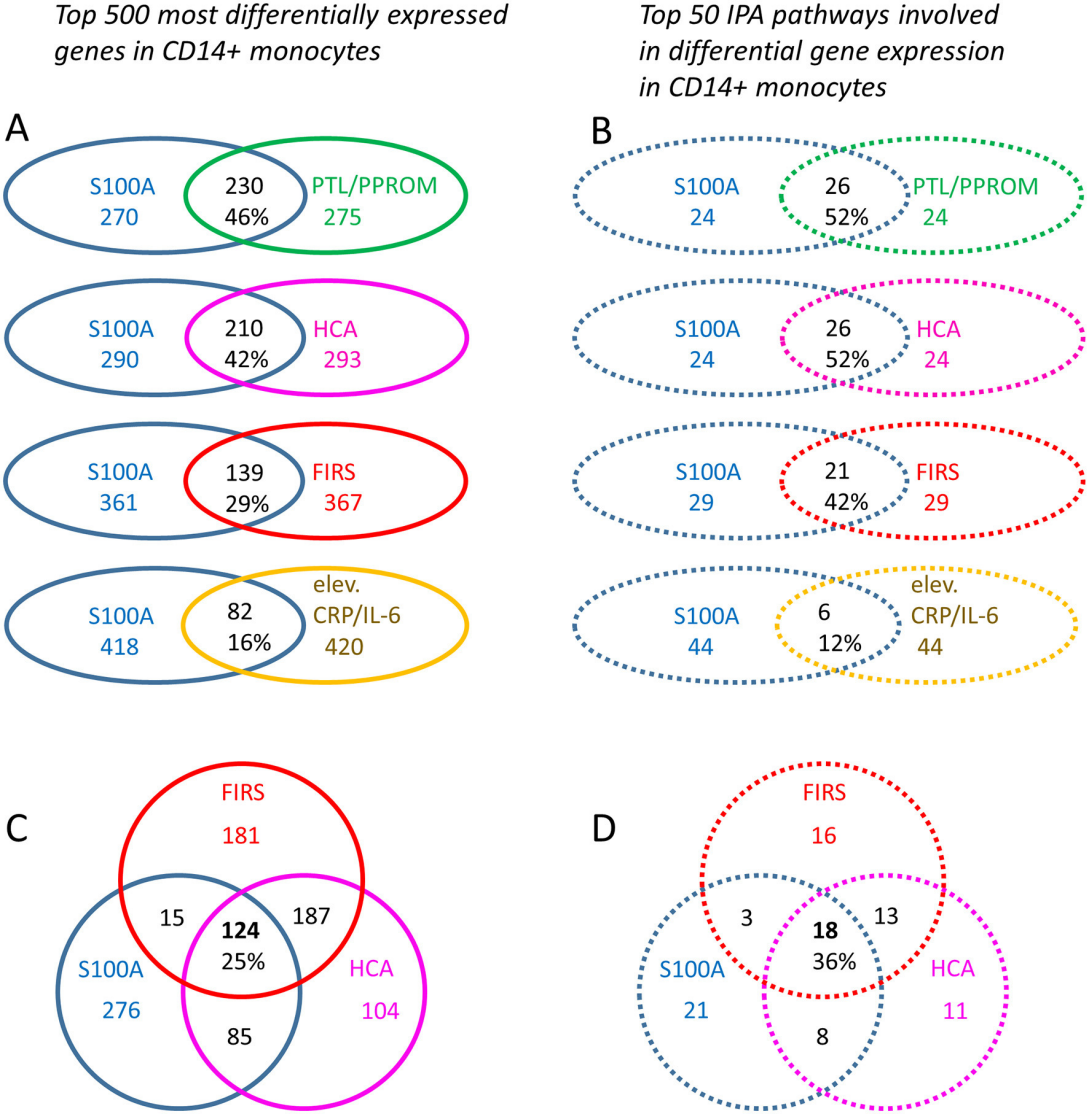
Differentially expressed monocyte genes and affected IPA pathways common to high monocyte S100A alarmin gene expression and clinical features associated with chorioamnionitis and a fetal inflammatory response in preterm infants. Top 500 differentially expressed (DE) genes (based on *p*-values) **(A)** and top 50 affected IPA pathways **(B)** were identified for each clinical grouping parameter. Common genes and pathways for the S100 high group in relation to HCA and FIRS are shown in **(C)** and **(D)**, respectively. When statistical significance was identical for the least regulated DE genes, a cut-off of exactly 500 could not be applied and numbers of DE genes may therefore differ slightly between groups. S100A high vs. low gene expression (500 genes, *p* < 0.0001, blue outline), presence of preterm labor (PTL)/preterm prelabor rupture of membranes (PPROM) (505 genes, *p* < 0.003, green outline); exposure to histological chorioamnionitis (HCA) (503 genes, *p* < 0.002, violet outline); histological fetal inflammatory response syndrome (FIRS) (502 genes, *p* < 0.0006, red outline); and elevated CRP/IL-6 in cord blood (506 genes, *p* < 0.013, yellow outline). The Venn diagrams demonstrate numbers and percentages for overlapping genes and pathways.

To obtain insight into possible mechanisms affecting preterm monocyte phenotype and function, we further analyzed differentially expressed genes and affected pathways in infants with high vs. low S100A gene expression under various clinical conditions. Out of 1936 DE genes (*q* < 0.049), top 500 DE genes were identified between the two S100A expression groups. Similarly, top 500 DE genes (based on *p*-values) were identified for clinical conditions indicative of inflammatory exposure, namely HCA vs. no HCA; FIRS vs. no FIRS, PTL/PPROM vs. physician-initiated delivery, and laboratory signs of early inflammation (elevated CRP/IL-6) vs. no such signs. When statistical significance was identical for the least regulated DE genes, a cut-off of exactly 500 could not be applied and numbers of DE genes therefore differ slightly between groups. Genes and pathways common to high S100A expression and the clinical conditions are demonstrated in Figure 7. In summary, 16–46% of ~500 top DE genes were common to high S100A groups and groups with different clinical features, with the largest overlap with PTL/PPROM and HCA (Figure 7A). A similar pattern was seen for top 50 regulated pathway with 12–52% of pathways common to high S100A groups and groups with different clinical features and the largest overlap was again seen with PTL/PPROM and HCA (Figure 7B). The weakest association in both cases was seen for high S100A group and elevated CRP/IL-6 at birth.

To narrow down the number of interesting genes and associated pathways, we excluded elevated CRP/IL-6 (low number of infants, poor overlap with S100A high group) and PTL/PPROM (large overlap with S100A high group but a significant number of infants also in the S100A low group). We conducted further analyses with the S100A high group in relation to HCA and FIRS, two clinical factors that are associated with inflammatory activation in the fetus as well as neonatal morbidity and outcome. Our analyses revealed that 124 DE genes and 18 affected pathways were common to all three groups (Figures 7C,D). Genes are listed in Table 5 and pathways with select references in Table 6. Further details for the 124 DE genes including fold change and fpkm levels are provided in Supplementary Table 4. In summary, affected pathways were largely inflammatory and associated with cytokine/chemokine signaling, chemotaxis and leukocyte trafficking as well as phagocytosis. Some of these 18 pathways were common to those affected by preterm vs. term birth, including pathways associated RhoA GTPases.

**Table 5:** Differentially expressed genes in cord blood monocytes common to high expression of S100A alarmins as well as HCA and FIRS (*n* = 124).

**Gene symbol**	**Official full name**	**Aliases**	**Corresponding protein function**
*ACSS2*	Acyl-CoA synthetase short chain family member 2	ACAS2, ACECS, ACS, ACSA, AceCS1, dJ1161H23.1	A cytosolic enzyme that catalyzes the activation of acetate for use in lipid synthesis and energy generation
*ACTR3*	Actin related protein 3	ARP3	A major constituent of the ARP2/3 complex
*ADAM10*	ADAM metallopeptidase domain 10	AD10, AD18, CD156c, CDw156, HsT18717, MADM, RAK, kuz	An ADAM family member that cleaves many proteins including TNF-alpha and E-cadherin
*ADCK3*	Coenzyme Q8A	COQ8A; ARCA2, CABC1, COQ10D4, COQ8, SCAR9	A mitochondrial protein similar to yeast ABC1, which functions in an electron-transferring membrane protein complex in the respiratory chain
*AKAP1*	A-kinase anchoring protein 1	AKAP21, AKAP149, AKAP84, D-AKAP1, PPP1R43, PRKA1, SAKAP84, TDRD17, AKAP1	A member of the AKAP family, binds to type I and type II regulatory subunits of PKA and anchors them to the mitochondrion
*ALOX5*	Arachidonate 5-lipoxygenase	5-LO, 5-LOX, 5LPG, LOG5	A member of the lipoxygenase gene family, plays a dual role in the synthesis of leukotrienes from arachidonic acid
*ASGR2*	Asialoglycoprotein receptor 2	ASGP-R2, ASGPR2, CLEC4H2, HBXBP, HL-2	A subunit of the asialoglycoprotein receptor
*ATP6V0D1*	ATPase H+ transporting V0 subunit d1	ATP6D, ATP6DV, P39, VATX, VMA6, VPATPD	A component of vacuolar ATPase (V-ATPase)
*B4GALT5*	Beta-1,4-galactosyltransferase 5	B4Gal-T5, BETA4-GALT-IV, beta4Gal-T5, beta4GalT-V, gt-V	Type II membrane-bound glycoproteins that appear to have exclusive specificity for the donor substrate UDP-galactose
*BATF*	Basic leucine zipper ATF-like transcription factor	B-ATF1, SFA-2, SFA2, BATF	A nuclear basic leucine zipper protein that belongs to the AP-1/ATF superfamily of transcription factors
*BTAF1*	B-TFIID TATA-box binding protein associated factor 1	MOT1, TAF(II)170, TAF172, TAFII170	A TAF (TATA box-binding protein-associated factor), which associates with TBP (TATA box-binding protein) to form the B-TFIID complex that is required for transcription initiation of genes by RNA polymerase II
*C17orf62*	Cytochrome b-245 chaperone 1	CYBC1, Eros	–
*C9orf84*	Shortage in chiasmata 1	SHOC1; ZIP2; MZIP2; ZIP2H	–
*CALM3*	Calmodulin 3	CALM, CAM1, CAM2, CAMB, CaM, CaMIII, HEL-S-72, PHKD, PHKD3	A member of a family of proteins that binds calcium and functions as a enzymatic co-factor
*CAMK2D*	Calcium/calmodulin dependent protein kinase II delta	CAMKD	A member of the serine/threonine protein kinase family and the Ca(2+)/calmodulin-dependent protein kinase subfamily
*CARS*	Cysteinyl-tRNA synthetase 1	CARS1, CYSRS, MGC:11246	A class 1 aminoacyl-tRNA synthetase, cysteinyl-tRNA synthetase
*CD177*	CD177 molecule	HNA-2a, HNA2A, NB1, NB1 GP, PRV-1, PRV1	A glycosyl-phosphatidylinositol (GPI)-linked cell surface glycoprotein that plays a role in neutrophil activation
*CD63*	CD63 molecule	LAMP-3, ME491, MLA1, OMA81H, TSPAN30	A member of the transmembrane 4 superfamily, also known as the tetraspanin family, the encoded protein is a cell surface glycoprotein that is known to complex with integrins
*CEACAM1*	CEA cell adhesion molecule 1	BGP, BGP1, BGPI	A member of the carcinoembryonic antigen (CEA) gene family, which belongs to the immunoglobulin superfamily, mediates cell adhesion via homophilic as well as heterophilic binding to other proteins of the subgroup
*CEACAM3*	CEA cell adhesion molecule 3	CD66D, CEA, CGM1, W264, W282	A member of the family of carcinoembryonic antigen-related cell adhesion molecules (CEACAMs)
*CFL1*	Cofilin 1	CFL, HEL-S-15, cofilin	An intracellular actin-modulating protein that binds and depolymerizes filamentous F-actin and inhibits the polymerization of monomeric G-actin in a pH-dependent manner
*CHMP2A*	Charged multivesicular body protein 2A	BC-2, BC2, CHMP2, VPS2, VPS2A	Protein belongs to the chromatin-modifying protein/charged multivesicular body protein (CHMP) family
*CNNM3*	Cyclin and CBS domain divalent metal cation transport mediator 3	ACDP3	–
*CPSF2*	Cleavage and polyadenylation specific factor 2	CPSF100	–
*CR1*	Complement C3b/C4b receptor 1 (Knops blood group)	C3BR, C4BR, CD35, KN	A member of the receptors of complement activation (RCA) family, a monomeric single-pass type I membrane glycoprotein, mediates cellular binding to particles and immune complexes that have activated complement
*CREB5*	cAMP responsive element binding protein 5	CRE-BPA, CREB-5, CREBPA	Belongs to the CRE (cAMP response element)-binding protein family containing zinc-finger and bZIP DNA-binding domains, functions as a CRE-dependent trans-activator
*CSF2RB*	Colony stimulating factor 2 receptor beta common subunit	CD131, CDw131, IL3RB, IL5RB, SMDP5, betaGMR	A common beta chain of the high affinity receptor for IL-3, IL-5, and CSF
*CYSTM1*	Cysteine rich transmembrane module containing 1	C5orf32, ORF1-FL49	–
*DOK3*	Docking protein 3	DOKL	–
*DYRK2*	Dual specificity tyrosine phosphorylation regulated kinase 2		Belongs to a family of protein kinases whose members are presumed to be involved in cellular growth and/or development
*EIF2AK4*	Eukaryotic translation initiation factor 2 alpha kinase 4	GCN2, PVOD2	A member of a family of kinases that phosphorylate the alpha subunit of eukaryotic translation initiation factor-2 (EIF2), resulting in the downregulaton of protein synthesis
*ERI1*	Exoribonuclease 1	3′HEXO, HEXO, THEX1	–
*EXOC6*	Exocyst complex component 6	EXOC6A, SEC15, SEC15L, SEC15L1, SEC15L3, Sec15p	Similar to the yeast gene product, which is essential for vesicular traffic from the Golgi apparatus to the cell surface, one of the components of a multiprotein complex required for exocytosis
*EXOC7*	Exocyst complex component 7	2-5-3p, BLOM4, EX070, EXO70, EXOC1, Exo70p, YJL085W	A component of the exocyst complex, is required for assembly of the exocyst complex and docking of the complex to the plasma membrane
*FAM109A*	PH domain containing endocytic trafficking adaptor 1	PHETA1; SES1; IPIP27A	A protein that localizes to the endosome and interacts with inositol polyphosphate 5-phosphatase OCRL-1
*FAM151B*	Family with sequence similarity 151 member B	UNQ9217	–
*FAM20A*	FAM20A golgi associated secretory pathway pseudokinase	AI1G, AIGFS, FP2747	A protein that is likely secreted and may function in hematopoiesis
*FCER1G*	Fc fragment of IgE receptor Ig	FCRG	A high affinity IgE receptor
*FCGR1A*	Fc fragment of IgG receptor Ia	CD64, CD64A, FCRI, IGFR1	A high-affinity Fc-gamma receptor
*FCGR1B*	Fc fragment of IgG receptor Ib	CD64b, FCG1, FCGR1, FCGR1A, FcRI, FcgammaRIa, IGFR1, IGFRB	A low affinity FcgammaRIB receptor that may play an important role in humoral immune response
*FCGR1C*	Fc fragment of IgG receptor Ic, pseudogene	FCGR1CP; CD64c; FCRIC; IGFR1; IGFRC	–
*FKBP1A*	FKBP prolyl isomerase 1A	FKBP-12, FKBP-1A, FKBP1, FKBP12, PKC12, PKCI2, PPIASE	A member of the immunophilin protein family, which play a role in immunoregulation and basic cellular processes involving protein folding and trafficking
*FNDC3B*	Fibronectin type III domain containing 3B	FAD104, PRO4979, YVTM2421	–
*FXN*	Frataxin	CyaY, FA, FARR, FRDA, X25	A mitochondrial protein which belongs to the FRATAXIN family, participates in regulating mitochondrial iron transport and respiration
*GBA*	Glucosylceramidase beta	GBA1, GCB, GLUC	A lysosomal membrane protein that cleaves the beta-glucosidic linkage of glycosylceramide
*GFRA2*	GDNF family receptor alpha 2	GDNFRB, NRTNR-ALPHA, NTNRA, RETL2, TRNR2	A member of the GDNF receptor family, a glycosylphosphatidylinositol(GPI)-linked cell surface receptor for GDNF and NTN, mediates activation of the RET tyrosine kinase receptor
*GK*	Glycerol kinase	GK1D, GK	Protein belongs to the FGGY kinase family, it is a key enzyme in the regulation of glycerol uptake and metabolism
*GNG5*	G protein subunit gamma 5		A member of a family of G proteins
*GPR84*	G protein-coupled receptor 84	EX33, GPCR4	–
*GTF2A2*	General transcription factor IIA subunit 2	HsT18745, T18745, TF2A2, TFIIA, TFIIA-12, TFIIA-gamma, TFIIAS	Factor for transcription initiation on TATA-containing class II genes
*GUSBP3*	GUSB pseudogene 3	GUSBP1, SMA3	–
*HCK*	HCK proto-oncogene, Src family tyrosine kinase	JTK9, p59Hck, p61Hck	A member of the Src family of tyrosine kinases. This protein is primarily hemopoietic, particularly in cells of the myeloid and B-lymphoid lineages
*HDAC1*	Histone deacetylase 1	GON-10, HD1, KDAC1, RPD3, RPD3L1	Belongs to the histone deacetylase/acuc/apha family and is a component of the histone deacetylase complex
*HSD3B7*	Hydroxy-delta-5-steroid dehydrogenase, 3 beta- and steroid delta-isomerase 7	CBAS1, PFIC4, SDR11E3	A member of the short-chain dehydrogenase/reductase superfamily
*IFNAR1*	Interferon alpha and beta receptor subunit 1	AVP, IFN-alpha-REC, IFNAR, IFNBR, IFRC	A type I membrane protein that forms one of the two chains of a receptor for interferons alpha and beta
*IGSF6*	Immunoglobulin superfamily member 6	DORA	–
*IL4R*	Interleukin 4 receptor	CD124, IL-4RAA, IL4R	Encodes the alpha chain of the interleukin-4 receptor, a type I transmembrane protein that can bind interleukin 4 and interleukin 13
*ITGB2*	Integrin subunit beta 2	CD18, LAD, LCAMB, LFA-1, MAC-1, MF17, MFI7	An integrin beta chain, which combines with multiple different alpha chains to form different integrin heterodimers
*JAK3*	Janus kinase 3	JAK-3_HUMAN, JAKL, L-JAK, LJAK, JAK3	A member of the Janus kinase (JAK) family of tyrosine kinases involved in cytokine receptor-mediated intracellular signal transduction
*KDM1A*	Lysine demethylase 1A	AOF2, BHC110, CPRF, KDM1, LSD1	A nuclear protein containing a SWIRM domain, a FAD-binding motif, and an amine oxidase domain. This protein silences genes by functioning as a histone demethylase
*KREMEN1*	Kringle containing transmembrane protein 1	ECTD13, KREMEN, KRM1	A high-affinity dickkopf homolog 1 (DKK1) transmembrane receptor that functionally cooperates with DKK1 to block wingless (WNT)/beta-catenin signaling
*L3MBTL3*	L3MBTL histone methyl-lysine binding protein 3	MBT-1, MBT1	A member of the malignant brain tumor (MBT) family of chromatin interacting transcriptional repressors, is associated with the repression of gene expression
*LAMTOR2*	Late endosomal/lysosomal adaptor, MAPK and MTOR activator 2	ENDAP, HSPC003, MAPBPIP, MAPKSP1AP, ROBLD3, Ragulator2, p14	Protein with suggested role in endosomal biogenesis
*LCP1*	Lymphocyte cytosolic protein 1	CP64, HEL-S-37, L-PLASTIN, LC64P, LPL, PLS2	A member of a family of actin-binding proteins plastins
*LGALS1*	Galectin 1	GAL1, GBP	A protein from a family of beta-galactoside-binding proteins implicated in modulating cell-cell and cell-matrix interactions
*LIMK2*	LIM domain kinase 2		Belongs to a small subfamily of LIM proteins with 2 N-terminal LIM motifs and a C-terminal protein kinase domain, phosphorylates cofilin, inhibiting its actin-depolymerizing activity
*LITAF*	Lipopolysaccharide induced TNF factor	PIG7, SIMPLE, TP53I7	Lipopolysaccharide-induced TNF-alpha factor, which is a DNA-binding protein and can mediate the TNF-alpha expression by direct binding to the promoter region of the TNF-alpha gene
*LMNB1*	Lamin B1	ADLD, LMN, LMN2, LMNB	B-type lamin protein, is a component of the nuclear lamina
*LUC7L*	LUC7 like	LUC7B1, Luc7, SR+89, hLuc7B1	–
*MAP3K14-AS1*	MAP3K14 antisense RNA 1		
*MAP4K1*	Mitogen-activated protein kinase kinase kinase kinase 1	HPK1	–
*MARVELD1*	MARVEL domain containing 1	GB14, MARVD1, MRVLDC1, bA548K23.8	–
*METTL7B*	Methyltransferase like 7B	ALDI	–
*MILR1*	Mast cell immunoglobulin like receptor 1	Allergin-1, C17orf60, MCA-32, MCA32	–
*MRPL28*	Mitochondrial ribosomal protein L28	MAAT1, p15	A 39S subunit protein, belongs to mitochondrial ribosomal proteins
*MTR*	5-methyltetrahydrofolate-homocysteine methyltransferase	HMAG, MS, cblG	5-methyltetrahydrofolate-homocysteine methyltransferase, catalyzes the final step in methionine biosynthesis
*MTRR*	5-methyltetrahydrofolate-homocysteine methyltransferase reductase	MSR, cblE	A member of the ferredoxin-NADP(+) reductase (FNR) family of electron transferases, functions in the synthesis of methionine by regenerating methionine synthase to a functional state
*MYO10*	Myosin X		A member of the myosin superfamily, represents an unconventional myosin
*MYO7B*	Myosin VIIB		Is involved in linking protocadherins to the actin cytoskeleton and is essential for proper microvilli function
*NCF4*	Neutrophil cytosolic factor 4	CGD3, NCF, P40PHOX, SH3PXD4	A cytosolic regulatory component of the superoxide-producing phagocyte NADPH-oxidase
*PART1*	Prostate androgen-regulated transcript 1	NCRNA00206	–
*PIK3AP1*	Phosphoinositide-3-kinase adaptor protein 1	BCAP	–
*PIK3IP1*	Phosphoinositide-3-kinase interacting protein 1	HGFL, TrIP, hHGFL(S)	–
*PIM1*	Pim-1 proto-oncogene, serine/threonine kinase	PIM	The protein encoded by this gene belongs to the Ser/Thr protein kinase family, and PIM subfamily. It plays a role in signal transduction in blood cells, contributing to both cell proliferation and survival
*PLB1*	Phospholipase B1	PLB, PLB/LIP	A membrane-associated phospholipase that displays lysophospholipase and phospholipase A2 activities through removal of sn-1 and sn-2 fatty acids of glycerophospholipids
*PLSCR1*	Phospholipid scramblase 1	MMTRA1B	–
*PLXNC1*	Plexin C1	CD232, PLXN-C1, VESPR	A member of the plexin family of transmembrane receptors for semaphorins
*POLR1E*	RNA polymerase I subunit E	PAF53, PRAF1	–
*PPM1M*	Protein phosphatase, Mg2+/Mn2+ dependent 1M	PP2C-eta, PP2CE, PP2Ceta	–
*PPP1R18*	Protein phosphatase 1 regulatory subunit 18	HKMT1098, KIAA1949	Protein phosphatase-1 interacts with regulatory subunits that target the enzyme to different cellular locations and change its activity toward specific substrates
*PSMB7*	Proteasome 20S subunit beta 7	Z	A member of the proteasome B-type family, it is a 20S core beta subunit in the proteasome
*PTPN2*	Protein tyrosine phosphatase non-receptor type 2	PTN2, PTPT, TC-PTP, TCELLPTP, TCPTP	A member of the protein tyrosine phosphatase (PTP) family known to be signaling molecules that regulate cell growth, differentiation, mitotic cycle, and oncogenic transformation
*RAB31*	RAB31, member RAS oncogene family	Rab22B	A small GTP-binding protein of the RAB family, participates in vesicle and granule targeting
*RGL4*	Ral guanine nucleotide dissociation stimulator like 4	Rgr	A protein similar to guanine nucleotide exchange factor Ral guanine dissociation stimulator
*RHBDD2*	Rhomboid domain containing 2	NPD007, RHBDL7	A member of the rhomboid family of membrane-bound proteases
*RHOG*	Ras homolog family member G	ARHG	A member of the Rho family of small GTPases, which cycle between inactive GDP-bound and active GTP-bound states and function as molecular switches in signal transduction cascades
*S100A11*	S100 calcium binding protein A11	HEL-S-43, MLN70, S100C	A member of the S100 family of proteins containing 2 EF-hand calcium-binding motifs
*SBNO2*	Strawberry notch homolog 2	KIAA0963, SNO, STNO	–
*SERPINA1*	Serpin family A member 1	A1A, A1AT, AAT, PI, PI1, PRO2275, alpha1AT, nNIF	A serine protease inhibitor whose targets include elastase, plasmin, thrombin, trypsin, chymotrypsin, and plasminogen activator
*SIPA1L2*	Signal induced proliferation associated 1 like 2	SPAL2, SPAR2	A member of the signal-induced proliferation-associated 1 like family containing a GTPase activating domain, a PDZ domain and a C-terminal coiled-coil domain with a leucine zipper
*SLC2A3*	Solute carrier family 2 member 3	GLUT3	–
*SNX20*	Sorting nexin 20	SLIC1	–
*SRF*	Serum response factor	MCM1	A member of the MADS box superfamily of transcription factors, stimulates both cell proliferation and differentiation
*SRP14*	Signal recognition particle 14	ALURBP	–
*ST3GAL2*	ST3 beta-galactoside alpha-2,3-sialyltransferase 2	Gal-NAc6S, SIAT4B, ST3GALII, ST3GalA.2	A type II membrane protein that catalyzes the transfer of sialic acid from CMP-sialic acid to galactose-containing substrates
*TCAIM*	T cell activation inhibitor, mitochondrial	TOAG1; TOAG-1; C3orf23	–
*TESC*	Tescalcin	CHP3, TSC	–
*TMBIM6*	Transmembrane BAX inhibitor motif containing 6	BAXI1, BI-1, TEGT	–
*TMEM117*	Transmembrane protein 117		–
*TMEM120A*	Transmembrane protein 120A	NET29, TMPIT	–
*TNFRSF1A*	TNF receptor superfamily member 1A	CD120a, FPF, TBP1, TNF-R, TNF-R-I, TNF-R55, TNFAR, TNFR1, TNFR55, TNFR60, p55, p55-R, p60	A member of the TNF receptor superfamily of proteins
*TPRKBP2*	TP53RK binding protein pseudogene 2		–
*TYSND1*	Trypsin domain containing 1	NET41	A protease that removes the N-terminal peroxisomal targeting signal (PTS2) from proteins produced in the cytosol, thereby facilitating their import into the peroxisome
*UBE2L3*	Ubiquitin conjugating enzyme E2 L3	E2-F1, L-UBC, UBCH7, UbcM4	A member of the E2 ubiquitin-conjugating enzyme family, participates in ubiquitination of proteins
*UFD1L*	Ubiquitin recognition factor in ER associated degradation 1	UFD1L	The encoded protein forms a complex with nuclear protein localization-4 and valosin-containing protein, and this complex is necessary for the degradation of ubiquitinated proteins
*URB1*	URB1 ribosome biogenesis homolog	C21orf108, NPA1	–
*WDFY3*	WD repeat and FYVE domain containing 3	ALFY, BCHS, MCPH18, ZFYVE25	A phosphatidylinositol 3-phosphate-binding protein that functions as a master conductor for aggregate clearance by autophagy
*WDFY3-AS1*	WDFY3 antisense RNA 1		ncRNA
*WDR59*	WD repeat domain 59	CDW12, FP977, p90-120	–
*VOPP1*	VOPP1 WW domain binding protein	ECOP, GASP, WBP1L2	–
*ZBTB41*	Zinc finger and BTB domain containing 41	FRBZ1, ZNF924	–
*ZNF337*	Zinc finger protein 337		A zinc finger domain containing protein
*ZNF438*	Zinc finger protein 438		–
*ZNF529*	Zinc finger protein 529		–

*Lists of differentially expressed genes were created using Qlucore Omics Explorer v3.4. Description of the genes (Gene Official Full Name, Aliases, info about corresponding protein transcript) are adapted from Gene ncbi database https://www.ncbi.nlm.nih.gov/gene/*.

**Table 6:** Canonical IPA pathways regulated in cord blood monocytes and common to high expression of S100A alarmins as well as HCA and FIRS (*n* = 18).

**Canonical pathways**	**Signaling pathway categories**	**Relation to inflammation and/or monocyte function (select references)**
fMLP Signaling in Neutrophils	Cellular Immune Response; Cytokine Signaling	
RhoGDI Signaling	Intracellular and Second Messenger Signaling	Involved in Rho-regulation, important for innate and adaptive immunity (35, 37)
STAT3 Pathway	Cellular Growth, Proliferation and Development; Transcriptional Regulation	Transcription activator, responds to cytokines and growth factors (45)
Production of Nitric Oxide and Reactive Oxygen Species in Macrophages	Cellular Immune Response	Plasma oxidative stress markers significantly increased in preterm infants (46)
Signaling by Rho Family GTPases	Intracellular and Second Messenger Signaling	Rho is a family of GTPases, important in innate and adaptive immunity (35)
Ephrin Receptor Signaling	Cell Morphology; Cellular Movement; Connective Tissue Development and Function	Ephrin proteins are involved in inflammation in vascular endothelium (41) including in monocytes (42)
Opioid Signaling Pathway	Neurotransmitters and Other Nervous System Signaling	N/A
CCR3 Signaling in Eosinophils	Cellular Immune Response; Cytokine Signaling	Important in leukocyte trafficking (47)
GNRH Signaling	Neurotransmitters and Other Nervous System Signaling	N/A
Protein Kinase A Signaling	Intracellular and Second Messenger Signaling	N/A
Breast Cancer Regulation by Stathmin1	Cancer; Disease-Specific Pathways	N/A
Rac Signaling	Intracellular and Second Messenger Signaling	Involved in monocyte migration (48)
Regulation of Actin-based Motility by Rho	Neurotransmitters and Other Nervous System Signaling	Rho is a family of GTPases, important in innate and adaptive immunity (35)
Chemokine Signaling	Cytokine Signaling; Organismal Growth and Development	
Fcy Receptor-mediated Phagocytosis in Macrophages and Monocytes	Cellular Immune Response	
Phospholipase C Signaling	Intracellular and Second Messenger Signaling	Chemotaxis in monocytes (49)
Synaptic Long Term Potentiation	Neurotransmitters and Other Nervous System Signaling	N/A
Phagosome Formation	Cellular Immune Response; Pathogen-Influenced Signaling	Important in innate and adaptive host defense against pathogens (50)

*Analysis of pathways was performed by Ingenuity Pathway Analysis (IPA) (Qiagen Bioinformatics). IPA signaling pathway categories are used to briefly describe each pathway. HCA, histological chorioamnionitis; FIRS, fetal inflammatory response syndrome with a fetal inflammatory response in placent*.

### Network Analysis Show That S100A8 and S100A9 Are Hub Genes in a Network Based on Genes Common to High S100A, FIRS and HCA and With Strong Correlation to Clinical Inflammatory Conditions

**Figure 8:**
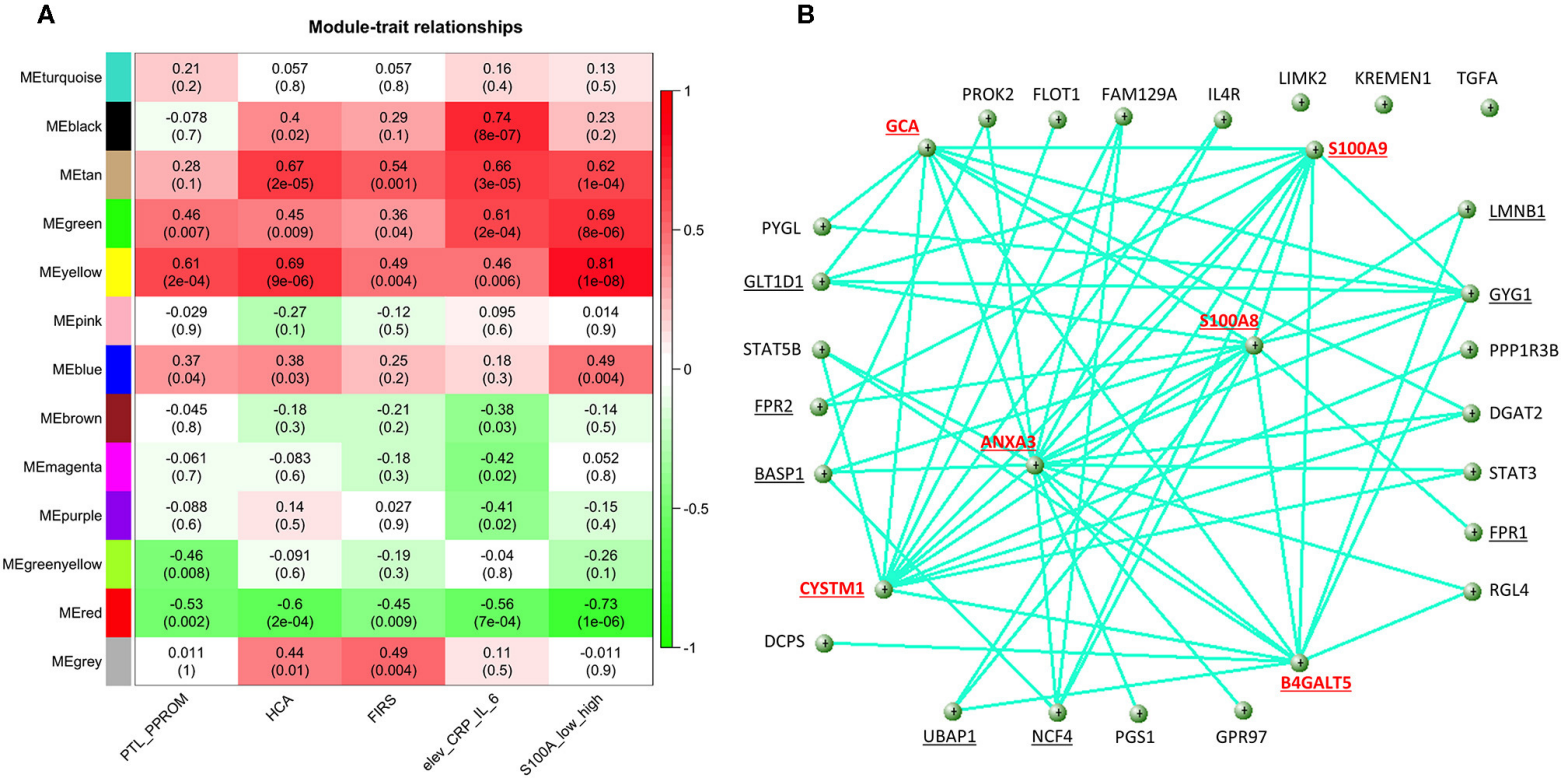
Results of weighted correlation network analysis of gene expression in cord blood monocytes from preterm infants. **(A)** The heatmap shows correlation and (significance) of the eigengenes of the modules in relation to S100A alarmin expression and clinical inflammatory conditions [preterm labor (PTL)/preterm prelabor rupture of membranes (PPROM); exposure to histological chorioamnionitis (HCA), histological fetal inflammatory response syndrome (FIRS), and elevated CRP/IL-6 in cord blood plasma]. Significant positive correlations for all traits were seen for green and yellow modules and negative correlations for the red module. **(B)** Visualization of the network for the top 30 hub genes in the yellow module (weight cutoff set from 0.19 to 1) with the most pronounced hub nodes marked in red and genes connected with S100A8 and S100A9 underlined.

For co-expression network analyses, we obtained 13 modules with sizes between 205 and 2,290 genes. The heatmap plot showing the correlation and significance of the eigengenes in relation to S100A expression and relevant clinical conditions is shown in Figure 8A. Of the modules identified, the red module was negatively correlated, and the green and the yellow modules were positively correlated with high S100A expression as well as PTL/PPROM, HCA, FIRS and elevated CRP/IL-6. In addition, 21 of the 124 DE genes we previously identified as common to FIRS, HCA and high S100A expression were identified as hub genes when considering all modules.

As S100A8, S100A9, and S100A12 genes were all found in the yellow module, this module was studied in further detail. The yellow module also showed the highest correlation with S100A expression in monocytes (Figure 8A). This module consisted of a total 836 genes and 41 of them were identified as hub genes within the module. The yellow module was to the greatest extent enriched with the 124 DE genes common to high S100A expression, HCA, and FIRS. 73/124 genes (58.9%) were found within the 836 genes of the module and 13/124 genes were among the 41 hub genes. S100A8 and S100A9 were identified as hub genes, while S100A12 was not.

Visualization of the network within the yellow module (Figure 8B) shows that S100A8 and S100A9 are major hubs with connectivities of 78 and 77, respectively. The other major hub nodes in this module are ANXA3, CYSTM1, B4GALT5, and GCA with connectivities of 98 96, 88, and 83, respectively. A list of hub genes for the yellow module is presented in Supplementary Table 5.

Gene Ontology enrichment for all the genes in the yellow module showed that these genes are highly relevant to inflammatory and leukocyte activation processes (Supplementary Table 6). The GO enrichment analysis made only for the hub genes showed similar enrichment with inflammation and immunity-related processes (data not shown).

## Discussion

In this study we found that gene expression of S100A alarmins in cord blood monocytes was associated with specific monocyte phenotypes characterized by their associations to inflammatory pathways and strong correlations to clinically relevant conditions. S100A alarmin genes were significantly up-regulated in preterm infants with exposure to chorioamnionitis, a fetal inflammatory response and gene expression changes associated with inflammatory pathways. In contrast, low S100A expression in preterm infants was associated with a clinical risk profile of SGA infants and a high proportion of physician-initiated deliveries.

In our first analyses, we found that gene expression in cord blood monocytes significantly differed between preterm and term infants and that the differences could largely be attributed to genes associated with inflammatory pathways. In addition to up-regulated genes and inflammatory proteins in the preterm vs. the term group, there was also a large variability of gene expression as well as inflammatory protein profiles within the preterm group, suggesting different patterns of inflammatory activation. This is in accordance with previous findings from global transcriptome analyses of cord blood monocytes where differences between term and preterm groups were accentuated in the presence of HCA in the preterm group (20). The number of genes that were differentially expressed between term and preterm infants was, however, 10-fold lower than in our study even when term and HCA-exposed preterm infants were compared (20). This may be explained by differences in analyses methods including the use of frozen and cultured vs. directly isolated monocytes in our study, but also by the fact that our study included a larger number of infants (33 preterm and 10 term vs. 11 preterm and 4 term) born at lower gestational ages (23–30 vs. 29–32 weeks) (20). The variable inflammatory protein profiles within the preterm group are also likely to reflect differences in prenatal exposure to infection or inflammation as previous studies show a significant association between inflammatory markers in cord blood and histological findings of HCA and, in particular, FIRS (10, 11, 51).

To identify monocyte gene expression associated with clinical and biochemical inflammatory signs within the preterm group, we investigated gene expression of alarmins S100A8, S100A9, and S100A12. We found an increased and highly correlated expression of all three genes in the preterm group with a variability suggesting association to different inflammatory patterns. In spite of a relatively small study group and an arbitrary cut-off based on median expression of S100A8 and S100A9 only, we found a strong correlation between S100A gene expression and clinical conditions that are associated with chorioamnionitis and fetal inflammation. As discussed below, there are numerous studies of S100A protein levels but, to our knowledge, this is the first study linking differences in monocyte S100A alarmin expression to a specific clinical inflammatory syndrome.

Identifying preterm infants with an increased risk of inflammation-related injury is of great importance in the clinical setting. Histological signs of HCA, and FIRS in particular, are associated with severe neonatal morbidities (12–14) as well as brain injury (11, 15) and neurodevelopmental sequels (11, 15). In addition, spontaneous onset of delivery has been related to preterm cerebral palsy (52) and elevated inflammatory markers in cord blood has been related not only to early onset sepsis but also to brain injury (11) and poor neurodevelopmental outcome (53). Characterizing the fetal immune response associated with exposure to chorioamnionitis and fetal inflammation is important to identify infants at risk for inflammatory injury and distinguish them from infants with other risk profiles and different mechanisms of injury. Remarkably, S100A8/A9 monocyte gene expression, with an arbitrary cut-off defined as above median of the preterm group, correctly identified 87% of HCA and 82% of FIRS exposed infants. In addition, all mothers in the S100A high group had spontaneous onset of delivery (PTL/PPROM) and all infants with clinically relevant elevation of CRP and/or IL-6 had high expression of S100A alarmins. Similarly, infants with the most pronounced pro-inflammatory protein profiles were found in the S100A high group. Equally important, infants with risk factors not related to inflammation (poor fetal growth, physician-initiated delivery due to maternal conditions or fetal compromise) were largely found in the low S100A group. This suggests that transcription of S100A defines a monocyte phenotype closely related to clinically defined risk factors.

In our study, single cases of HCA and FIRS were not correctly classified by S100A gene expression. This may be explained by the choice of cut-off value or by factors related to classification and severity of the intrauterine infection/inflammation. We have not related our findings to severity of placenta inflammation due to a limited number of patients, and without access to amniotic fluid it cannot be determined if there was intra-amniotic infection without placenta findings, or whether the inflammatory process was infectious or sterile (5, 54).

There are a number of factors apart from chorioamnionitis that could potentially affect the inflammatory response in cord monocytes in our preterm infants. Gestational age was significantly lower in the S100A high group, but differences between groups remained after statistical correction for gestational age at birth. Labor and vaginal delivery is in itself associated with an inflammatory response in the fetus (55, 56), and spontaneous onset of delivery was significantly more common in the group with high S100A gene expression. There was, however, a significant number of infants with spontaneous onset of delivery also in the S100A low group, and in addition, cesarian section and the frequency of vaginal delivery did not significantly differ between groups with or without spontaneous onset of labor. In addition, the term group included only infants born after vaginal delivery, and all infants had low expression of S100A alarmins. These findings suggest that labor or way of delivery could not in itself explain increased S100A gene expression. There is also a risk that fetal monocyte response could be related to infection or inflammation in the mother, not related to chorioamnionitis. In our study, we identified mothers with fever and/or elevated CRP that were clinically diagnosed and treated as chorioamnionitis and found that symptoms in the mothers did not differ between groups with high or low S100A gene expression. Comprehensive studies show that most cases of verified HCA are subclinical and that a significant number of “clinical chorioamnionitis” is unrelated to intrauterine infection or inflammation (6). In our study, fetal S100A monocyte expression had a stronger association with definite histological signs than with unspecific clinical symptoms.

Based on our findings of S100A monocyte gene upregulation in association with clinical signs of inflammation, we investigated if S100A8 and S100A9 proteins in cord blood could serve as markers for exposure to chorioamnionitis and fetal inflammation. We found that plasma levels of both proteins were increased in term vs. preterm infants and in association with HCA, FIRS and spontaneous onset of delivery (PTL/PPROM) within the preterm group. Elevated levels of the S100A8/A9 protein dimer has previously been described in healthy term infants compared with preterm infants in the absence of exposure to chorioamnionitis or signs of infection (19). In preterm infants, lower S100A8/A9 protein levels are associated with an *increased* risk for late onset sepsis (19), while another study shows that HCA and FIRS are associated with a *decreased* risk for late onset sepsis in preterm infants (18). Our novel findings of elevated S100A8 and S100A9 proteins in association with HCA and FIRS may provide a link between these findings, but further studies are needed to confirm such a connection. The S100A8/A9 protein dimer is elevated in neonatal sepsis in preterm infants (28), but to our knowledge, only one study has previously studied S100A8/A9 in preterm cord blood in association with chorioamnionitis and no changes were found (29). In that study, diagnosis of chorioamnionitis was based on clinical signs only, and proteins were analyzed in supernatant from whole blood cultures which may explain the differing findings (29).

In our study, high expression of S100A8 and S100A9 genes in cord blood monocytes was accompanied by elevated protein levels within the preterm group, which is contrary to the inverse relation seen in term infants. The inverse relation in term infants suggest that cells other than monocytes, most likely neutrophils, are the main source of S100A proteins in this group. S100A8 and S100A9 represents ~5% of cytosolic proteins in monocytes, but nearly half of cytosolic proteins in neutrophils (23). Neutrophils may also be of importance as a protein source in preterm infants under inflammatory conditions. While neutrophils of maternal origin are found in the placenta during HCA (57), fetal neutrophils invading umbilical cord is the hallmark of FIRS (7). Neutrophils of fetal as well as maternal origin are found in amniotic fluid in association with intraamniotic infection/inflammation and extremely preterm birth (58, 59). Neutrophils also release S100A alarmins under other inflammatory conditions in the preterm infant (60). In addition, elevated proteins S100A8, S100A9, and S100A12 have been identified as biomarkers for intra-amniotic infection and inflammation (61, 62) and, as reviewed by Buhimschi et al. (62), a panel of amniotic fluid proteins including S100A8 and S100A12 predicts clinically relevant HCA and FIRS as well as early onset sepsis and elevated inflammatory proteins in cord blood in preterm infants. Furthermore, increased gene expression of alarmins S100A8, A9, and A12 is found in umbilical cord tissue from preterm infants with FIRS and GO enrichment analysis show associations with neutrophil extravasation as well as monocyte migration (63). These findings suggest that fetal cells, other than circulating monocytes could be the source of proteins S100A8 and S100A9 released into the fetal blood stream also in the preterm infant. The positive correlation between high monocyte S100A gene expression and elevated protein levels in preterm infants under inflammatory conditions suggests, however, that monocytes may contribute to the increase in S100A protein levels, either by direct release of proteins or by inducing protein release from other cell types. As discussed below, S100A8/A9 proteins in cord blood could affect monocyte phenotype, and it is also possible that increased expression of S100A alarmins in cord tissue and increased levels of S100A8/A9 plasma proteins precede and contribute to monocyte phenotype changes rather than being caused by increased monocyte gene expression.

We found that close to 2,000 genes were differentially regulated in monocytes with either high or low expression of S100A8 and S100A9 genes. This was at the same level as number of genes differentially regulated between term and preterm infants. It is likely that this pronounced difference in gene expression pattern is associated with different functional monocyte phenotypes. Preterm monocytes are commonly characterized by a hypo-responsive phenotype with attenuated pro-inflammatory gene response (64). Exposure to HCA accentuates this pattern with reduced expression of key inflammatory genes associated with TLR activation and results in an attenuated response to Staphylococcus epidermis challenge (20). These findings suggest that exposure to chorioamnionitis may alter monocyte phenotype and later susceptibility to infections (20) and explain the decreased risk of late onset sepsis in preterm infants exposed to HCA (18). Recent studies have also suggested a role for S100A8/A9 protein dimer in induction of a hypo-responsive phenotype (19). Monocytes from healthy term infants demonstrate a hypo-responsive phenotype when compared with adult monocytes, and this phenotype is induced by high levels of S100A8/A9 in term cord blood (19, 27). The altered phenotype is explained by an altered transcription of genes in TRIF and MyD88 associated pathways following TLR4 activation by the S100A alarmins. The resulting imbalance leads to an attenuated cytokine response with decreased risk for hyperinflammation but with adequate pathogen defense and a decreased risk for sepsis (19). Preterm infants had generally lower levels of S100A8/A9 in cord blood than term infants and low levels within the preterm group were associated with an increased risk for sepsis (19). No infants with exposure to chorioamnionitis or with laboratory signs of fetal inflammation were included in this study (19).

In our study, preterm infants had lower levels of proteins S100A8 and S100A9 compared to term infants as found in previous studies (19), but HCA and FIRS resulted in significantly elevated concentrations within the preterm group with levels similar to those seen in term infants. It is thus possible that S100A8/A9 could alter monocyte phenotype in infants exposed to FIRS as in term infants (19) and help explain the transcriptional differences regarding S100A alarmins. However, none of the key down-regulated genes in HCA-exposed monocytes (20) or TLR4-related genes associated with S100A8/A9 immune-programming (19) were among the 124 genes that were differentially expressed in association with high S100A8/A9 gene expression as well as FIRS and HCA in our study. The connection between clinical inflammatory conditions, S100A8/A9 protein activity and monocyte phenotype warrants further investigation.

The monocyte hypo-responsiveness previously described in association with exposure to HCA (18, 20) and implied by increased levels of S100A8/A9 proteins in association with HCA and FIRS in our study is difficult to link to neonatal morbidities associated with HCA and FIRS. However, elevated S100A8/A9 are normalized within a week after birth in term infants (27) and experimental studies show that early endotoxin tolerance induced in fetal sheep is followed by an accentuated inflammatory monocyte response (65). It is thus possible that an early hypo-responsiveness induced by FIRS could be combined with a delayed, and potentially harmful hyper-inflammation. Longitudinal studies of monocyte phenotype and changes in S100A8/A9 protein levels in preterm infants are needed.

The most important reason to investigate monocyte phenotypes in preterm infants is to find associations with early morbidities that will help us identify underlying mechanisms of inflammation-induced injury. In our study, only the proportion of infants with patent ductus arteriosus differed between S100A high and low groups. An association between patent ductus arteriosus and chorioamnionitis is previously described (13). No differences for other morbidities were seen. Possible explanations are small study groups, few infants with severe disease and high risk infants with poor fetal growth within the S100A low group. It is, however, notable that the only case of early onset sepsis was found in the S100A high group and the only two cases of late onset sepsis in the S100A low group.

Pathway analysis based on genes that were differentially expressed in the S100A high group as well as FIRS and HCA groups identified several pathways that warrant further investigation. Cytokine/chemokine signaling and pathways associated with phagocytosis and oxygen free radical formation were, not unexpectedly, affected as these functions are well-described in monocytes in response to infectious and inflammatory challenges. Less well-described in monocytes than in other innate immune cells such as neutrophils and macrophages, are the small GTPases RhoA and RAC. These GTPases regulate the remodeling of actin cytoskeleton that is required for immune cell functions such as migration and phagocytosis (35, 48). Notably, 4/13 inflammation-related pathways were directly associated with RhoA and RAC signaling and at least one other more loosely [STAT3 signaling (66)]. RhoA GTPase signaling pathways have been implicated in preterm births (67), but have, to our knowledge, not been studied in cord blood monocytes or in association with chorioamnionitis.

Network analysis revealed that S100A8 and S100A9 are hub nodes in a network strongly related to clinical inflammatory conditions. Gene ontology enrichment analyses of genes within this network confirmed a strong relation to inflammatory and immune-related mechanisms within the monocytes and confirmed previous finding in analyses of genes and pathways common to high S100A expression, HCA and FIRS. Our findings suggest that S100A alarmins are not only responder genes but may be central in networks associated with specific monocyte phenotypes.

S100A8 and S100A9 were among the genes with the highest number of connectivities. In contrast, S100A12 was found within the same network but was not identified as a hub gene. This suggests that, in spite of very strong co-expression with S100A8 and S100A9, alarmin S100A12 may have other functions in relation to monocyte phenotype and outcome.

Network analysis also revealed connections between S100A8 and S100A9 and other major hub nodes that may be important in mechanisms related to monocyte function. This is exemplified by the hub gene ANXA3, that was also among the 500 DE genes in monocytes with high or low S100A expression. Annexin 3 is found in myeloid cells in specific granules that are translocated upon cell activation (68) and interactions between the annexin family proteins and S100A proteins have previously been described (69). ANXA3 is also upregulated together with S100A9 and S100A12 in peripheral blood in children with severe infections (70). These findings suggest that interactions between annexin 3 and S100A alarmins in relation to monocyte function and immune activation warrant further investigation.

In summary, we show that gene expression of S100A alarmins in cord blood monocytes was significantly up-regulated in preterm compared with term infants. A high expression of S100A alarmins within the preterm group was accompanied by pronounced changes in overall gene expression and was strongly associated with spontaneous onset of delivery, HCA, FIRS and a pro-inflammatory protein profile. This implies a direct link between exposure to chorioamnionitis and an altered monocyte phenotype characterized by high expression of S100A8 and S100A9 genes. These findings were supported by differential gene expression and network analyses showing a strong association between S100A alarmin expression and inflammation-associated pathways in the preterm infant. We also show that S100A8 and S100A9 proteins were elevated in cord blood plasma from preterm infants with high monocyte S100A alarmin gene expression and inflammatory conditions, to levels resembling those seen in term infants. Our study is, however, limited by a small sample size and the exploratory nature of the study using complex methods aimed at characterizing monocyte phenotypes. Our findings may help to identify clinically relevant markers (not limited to S100A proteins) to identify high-risk infants in the future, but this warrants further extensive studies in larger patient groups. In addition, the connection between elevated protein levels, monocyte phenotype and risk for inflammation-associated morbidities warrants further investigation as changes in monocyte gene expression may also be important for the adaptation of preterm infants to a postnatal life with additional inflammatory and infectious challenges. Furthermore, our study is limited to clinical findings and additional mechanistic studies are needed. A deeper understanding of immune regulation in the compromised preterm infants may help us clarify mechanisms of injury and identify infants for potential therapeutic interventions.

## Data Availability Statement

The raw data supporting the conclusions of this article will be made available by the authors, without undue reservation, to any qualified researcher.

## Ethics Statement

The studies involving human participants were reviewed and approved by Regional Ethic's Committee at the Sahlgrenska University Hospital, Gothenburg; application (EPN Gbg 933-16, T350-18). Written informed consent to participate in this study was provided by the participants' legal guardian/next of kin.

## Author Contributions

This study was conceived and designed by VG, CM, and KS. Clinical sampling and experiments were performed by VG, HP, I-MF, BJ, AH, and KS. Results were analyzed by VG, HN, HR, and KS. VG, HN, CM, and KS drafted the manuscript. All authors critically reviewed and edited the work.

## Conflict of Interest

The authors declare that the research was conducted in the absence of any commercial or financial relationships that could be construed as a potential conflict of interest.
