# Supplementary material for: Expression of S100A Alarmins in Cord Blood Monocytes Is Highly Associated With Chorioamnionitis and Fetal Inflammation in Preterm Infants
Source: Front Immunol. 2020 Jun 16;11:1194. doi: 10.3389/fimmu.2020.01194 (PMC7308505; doi:10.3389/fimmu.2020.01194)
Supplement: Supplementary file 1 [file Table_1.DOCX]

***Supplementary Table 1. Analyses of inflammatory proteins in cord blood by BioPlex Elisa in preterm (n=33) vs term (n=10) infants.***

|  | **protein name** | **geom.mean (log2 mean±SD)** | **geom.mean (log2 mean±SD)** | **q-value** | **p-value** |
| --- | --- | --- | --- | --- | --- |
| ***Preterm vs term infants*** | | | | | |
|  |  | ***Preterm*** | ***Term*** |  |  |
| Significant. q≤0.049 | MMP-1 | 1251.71  (10.29±1.27) | 107.49  (6.75±0.60) | 0.0000 | 0.0000 |
|  | SCF | 515.56  (9.01±0.73) | 170.54  (7.41±0.34) | 0.0000 | 0.0000 |
|  | s-TNF-R1 | 3150.25  (11.62±0.79) | 926.73  (9.86±0.48) | 0.0000 | 0.0000 |
|  | sTNF-R2 | 676.47  (9.40±0.81) | 219.79  (7.78±0.32) | 0.0000 | 0.0000 |
|  | sCD30/TNFRSF8 | 944.32  (9.88±0.91) | 269.66  (8.08±0.43) | 0.0000 | 0.0000 |
|  | M-CSF | 56.36  (5.82±0.70) | 21.95  (4.46±0.36) | 0.0000 | 0.0000 |
|  | CTACK | 226.81  (7.83±0.58) | 526.03  (9.04±0.59) | 0.0000 | 0.0000 |
|  | sCD163 | 164723.57  (17.33±1.08) | 42318.47  (15.37±0.41) | 0.0000 | 0.0000 |
|  | MCP-1(MCAF) | 201.94  (7.66±1.82) | 21.05  (4.40±0.59) | 0.0000 | 0.0000 |
|  | APRIL/TNFSF13 | 312284.19  (18.25±0.65) | 142539.28  (17.12±0.28) | 0.0000 | 0.0000 |
|  | Osteopontin (OPN) | 52362.63  (15.68±1.21) | 12503.12  (13.61±0.30) | 0.0000 | 0.0000 |
|  | IFN-b | 52.81  (5.72±0.67) | 26.30  (4.72±0.25) | 0.0002 | 0.0000 |
|  | Chitinase 3-like 1 | 20214.81  (14.30±0.76) | 9371.08  (13.19±0.31) | 0.0004 | 0.0001 |
|  | TNF-b | 3.69  (1.88±2.09) | 0.34  (-1.576±2.44) | 0.0005 | 0.0001 |
|  | gp130/sIL-6Rb | 39188.02  (15.26±1.19) | 12529.14  (13.61±0.33) | 0.0005 | 0.0001 |
|  | IL-8 | 134.71  (7.07±2.18) | 16.51  (4.05±1.13) | 0.0007 | 0.0001 |
|  | IL-35 | 81.29  (6.35±2.94) | 5.54  (2.47±0.00) | 0.0007 | 0.0002 |
|  | IL-15 | 44.78  (5.49±3.36) | 2.35  (1.23±0.00) | 0.0012 | 0.0003 |
|  | SCGF-b | 145622.63  (17.15±0.54) | 86956.12  (16.41±0.49) | 0.0012 | 0.0003 |
|  | IL-7 | 1.19  (0.26±2.58) | 11.15  (3.48±0.45) | 0.0012 | 0.0003 |
|  | TSLP | 26.28  (4.72±0.83) | 12.76  (3.67±0.21) | 0.0012 | 0.0003 |
|  | Pentraxin-3 | 26621.65  (14.70±1.70) | 6229.93  (12.61±0.38) | 0.0013 | 0.0004 |
|  | MIF | 7508.84  (12.87±1.22) | 2599.47  (11.34±0.45) | 0.0014 | 0.0004 |
|  | MIP-1a | 5.54  (2.47±1.14) | 2.11  (1.08±0.28) | 0.0015 | 0.0005 |
|  | IFN-g | 75.37  (6.24±1.63) | 19.20  (4.26±0.26) | 0.0015 | 0.0005 |
|  | sIL-6Ra | 6620.90  (12.69±1.38) | 2073.71  (11.02±0.39) | 0.0015 | 0.0005 |
|  | MMP-2 | 41136.32  (15.33±1.37) | 13354.15  (13.71±0.51) | 0.0020 | 0.0007 |
|  | IL-34 | 18.22  (4.19±1.43) | 5.61  (2.49±0.81) | 0.0026 | 0.0009 |
|  | IL-5 | 26.31  (4.72±2.71) | 2.84  (1.51±2.30) | 0.0043 | 0.0016 |
|  | TRAIL | 61.93  (5.95±0.47) | 89.76  (6.49±0.45) | 0.0060 | 0.0023 |
|  | G-CSF | 459.35  (8.84±1.69) | 136.81  (7.10±0.44) | 0.0062 | 0.0025 |
|  | MMP-3 | 7930.11  (12.95±0.68) | 5184.54  (12.34±0.21) | 0.0175 | 0.0073 |
|  | VEGF | 199.77  (7.64±1.10) | 95.21  (6.57±0.98) | 0.0208 | 0.0089 |
|  | IL-12(p40) | 175.31  (7.45±0.94) | 102.39  (6.68±0.31) | 0.0317 | 0.0140 |
|  | IL-26 | 827.25  (9.69±0.33) | 687.40  (9.43±0.17) | 0.0409 | 0.0186 |
| *Not significant. q>0.049* | *IL-20* | *37.80  (5.24±0.89)* | *23.62  (4.56±0.48)* | *0.0562* | *0.0263* |
|  | *IL-1ra* | *1452.55  (10.50±2.83)* | *365.57  (8.51±0.56)* | *0.0663* | *0.0324* |
|  | *IL-22* | *25.20  (4.66±2.01)* | *8.77  (3.13±1.44)* | *0.0663* | *0.0327* |
|  | *IL-6* | *39.46  (5.30±3.78)* | *6.05  (2.60±1.61)* | *0.0682* | *0.0345* |
|  | *IL-2* | *43.95  (5.46±0.99)* | *27.76  (4.80±0.29)* | *0.0823* | *0.0427* |
|  | *Osteocalcin* | *3252.88  (11.67±1.22)* | *1834.28  (10.84±0.69)* | *0.0865* | *0.0461* |
|  | *TWEAK/TNFSF12* | *149.18  (7.22±0.86)* | *100.15  (6.65±0.34)* | *0.0973* | *0.0531* |
|  | *Eotaxin* | *25.54  (4.67±0.95)* | *38.61  (5.27±0.52)* | *0.1207* | *0.0698* |
|  | *IL-32* | *2.34  (1.23±2.21)* | *0.95  (-0.08±0.00)* | *0.1207* | *0.0705* |
|  | *LIGHT/TNFSF14* | *4.17  (2.06±2.55)* | *1.37  (0.45±1.66)* | *0.1207* | *0.0682* |
|  | *IL-2Ra* | *110.23  (6.78±0.62)* | *85.57  (6.42±0.41)* | *0.1355* | *0.0810* |
|  | *MIG* | *498.76  (8.96±0.46)* | *419.35  (8.71±0.17)* | *0.1667* | *0.1017* |
|  | *GRO-a* | *741.54  (9.53±1.22)* | *473.76  (8.89±0.12)* | *0.1679* | *0.1047* |
|  | *TNF-a* | *48.80  (5.61±0.61)* | *39.45  (5.30±0.13)* | *0.1909* | *0.1215* |
|  | *MCP-3* | *1.42  (0.50±2.68)* | *3.33  (1.73±0.60)* | *0.2389* | *0.1551* |
|  | *IL-11* | *30.20  (4.92±1.21)* | *20.00  (4.32±1.33)* | *0.2941* | *0.1948* |
|  | *GM-CSF* | *1.55  (0.63±2.40)* | *0.76  (-0.40±1.61)* | *0.3273* | *0.2211* |
|  | *IL-10* | *6.11  (2.61±1.62)* | *3.87  (1.95±1.33)* | *0.3639* | *0.2504* |
|  | *IL-4* | *0.87  (-0.21±1.85)* | *1.37  (0.45±0.42)* | *0.3846* | *0.2697* |
|  | *IL28A* | *92.87  (6.54±0.62)* | *80.00  (6.32±0.23)* | *0.4012* | *0.2866* |
|  | *IL-17* | *9.55  (3.26±0.61)* | *11.02  (3.46±0.31)* | *0.4068* | *0.2959* |
|  | *IL-29* | *64.57  (6.01±2.31)* | *38.16  (5.25±1.82)* | *0.4626* | *0.3425* |
|  | *IFN-a2* | *17.43  (4.12±0.78)* | *14.78  (3.89±0.36)* | *0.4934* | *0.3717* |
|  | *MIP-1b* | *168.42  (7.40±0.39)* | *156.61  (7.29±0.22)* | *0.5559* | *0.4260* |
|  | *b-NGF* | *1.07  (0.09±2.53)* | *0.67  (-0.578±1.75)* | *0.5642* | *0.4396* |
|  | *SDF-1a* | *922.28  (9.85±0.44)* | *990.49  (9.95±0.22)* | *0.6256* | *0.4956* |
|  | *HGF* | *774.54  (9.60±1.21)* | *654.84  (9.36±0.43)* | *0.6694* | *0.5479* |
|  | *IL-1a* | *8.23  (3.04±1.49)* | *10.04  (3.33±0.65)* | *0.6694* | *0.5563* |
|  | *RANTES* | *8190.23  (13.00±1.09)* | *9436.26  (13.20±0.53)* | *0.6694* | *0.5559* |
|  | *IL-1b* | *1.79  (0.84±1.63)* | *1.46  (0.54±0.30)* | *0.6840* | *0.5774* |
|  | *IL-16* | *151.95  (7.25±2.56)* | *111.89  (6.81±1.76)* | *0.7193* | *0.6165* |
|  | *IL-9* | *217.43  (7.76±0.43)* | *226.91  (7.83±0.25)* | *0.7564* | *0.6604* |
|  | *LIF* | *2.18  (1.12±3.37)* | *1.49  (0.58±3.29)* | *0.7564* | *0.6679* |
|  | *IL-12(p70)* | *4.21  (2.07±0.93)* | *3.90  (1.97±0.46)* | *0.8365* | *0.7496* |
|  | *FGF basic* | *34.57  (5.11±0.87)* | *36.25  (5.18±0.33)* | *0.8685* | *0.8008* |
|  | *IL-18* | *34.86  (5.12±0.88)* | *33.31  (5.06±0.58)* | *0.8685* | *0.7993* |
|  | *BAFF/TNFSF13B* | *10995.97  (13.42±1.11)* | *11323.25  (13.47±0.64)* | *0.9324* | *0.9080* |
|  | *IL-13* | *2.36  (1.24±1.04)* | *2.40  (1.27±0.35)* | *0.9324* | *0.9203* |
|  | *IL-19* | *33.22  (5.05±2.11)* | *31.17  (4.96±0.69)* | *0.9324* | *0.9006* |
|  | *IL-27 (p28)* | *28.14  (4.81±2.59)* | *26.15  (4.71±0.66)* | *0.9324* | *0.9062* |
|  | *IP-10* | *540.14  (9.08±1.43)* | *520.95  (9.03±1.08)* | *0.9324* | *0.9104* |
|  | *PDGF-bb* | *889.32  (9.80±1.24)* | *868.87  (9.76±0.64)* | *0.9522* | *0.9522* |
