# Supplementary material for: Expression of S100A Alarmins in Cord Blood Monocytes Is Highly Associated With Chorioamnionitis and Fetal Inflammation in Preterm Infants
Source: Front Immunol. 2020 Jun 16;11:1194. doi: 10.3389/fimmu.2020.01194 (PMC7308505; doi:10.3389/fimmu.2020.01194)
Supplement: Supplementary file 2 [file Table_2.DOCX]

***Supplementary Table 2. Analyses of inflammatory proteins in cord blood by BioPlex Elisa in preterm infants with high (n=17) or low S100A (n=16) alarmin gene expression in cord blood monocytes.***

|  | **protein name** | **geom.mean (log2 mean±SD)** | **geom.mean (log2 mean±SD)** | **q-value** | **p-value** |
| --- | --- | --- | --- | --- | --- |
| ***High/low expression of S100A8 and S100A9 in monocytes*** | | | | | |
|  |  | ***High S100A8/A9*** | ***Low S100A8/A9*** |  |  |
| Significant. q≤0.049 | G-CSF | 986.30  (9.95±1.55) | 193.21  (7.60±0.64) | 0.0004 | 0.0000 |
|  | GRO-a | 1074.47  (10.07±1.41) | 487.07  (8.93±0.51) | 0.0407 | 0.0050 |
|  | IFN-g | 123.39  (6.95±1.52) | 43.11  (5.43±1.39) | 0.0407 | 0.0053 |
|  | IL-1b | 2.90  (1.54±1.85) | 1.04  (0.05±0.85) | 0.0473 | 0.0071 |
|  | IL-1ra | 3666.02  (11.84±2.33) | 508.70  (8.99±2.62) | 0.0354 | 0.0026 |
|  | IL-11 | 44.25  (5.47±1.22) | 19.58  (4.29±0.87) | 0.0401 | 0.0036 |
|  | IL-13 | 3.20  (1.68±0.84) | 1.67  (0.74±1.05) | 0.0473 | 0.0074 |
|  | IL-6 | 174.07  (7.44±3.74) | 7.34  (2.88±1.95) | 0.0040 | 0.0002 |
|  | IL-8 | 259.79  (8.02±2.27) | 64.00  (6.00±1.53) | 0.0407 | 0.0052 |
|  | LIF | 6.48  (2.70±3.54) | 0.63  (-0.66±2.11) | 0.0354 | 0.0028 |
|  | MIP-1a | 8.69  (3.12±1.07) | 3.32  (1.73±0.68) | 0.0040 | 0.0001 |
|  | M-CSF | 68.79  (6.10±0.69) | 44.96  (5.49±0.57) | 0.0487 | 0.0082 |
|  | TNF-a | 59.59  (5.90±0.61) | 38.91  (5.28±0.43) | 0.0354 | 0.0025 |
| *Not significant. q>0.049* | *APRIL/TNFSF13* | *341974.54  (18.38±0.54)* | *281739.05  (18.10±0.75)* | *0.2921* | *0.1935* |
|  | *BAFF/TNFSF13B* | *13912.73  (13.76±1.04)* | *8422.31  (13.04±1.10)* | *0.1435* | *0.0648* |
|  | *b-NGF* | *2.18  (1.13±1.79)* | *0.47  (-1.08±2.78)* | *0.0513* | *0.0107* |
|  | *Chitinase 3-like 1* | *22991.67  (14.49±0.61)* | *17470.90  (14.09±0.88)* | *0.1973* | *0.1178* |
|  | *CTACK* | *215.53  (7.75±0.37)* | *240.30  (7.91±0.76)* | *0.4160* | *0.3350* |
|  | *Eotaxin* | *22.55  (4.50±0.94)* | *29.41  (4.88±0.96)* | *0.3229* | *0.2283* |
|  | *FGF basic* | *41.39  (5.37±0.82)* | *28.19  (4.82±0.85)* | *0.1467* | *0.0686* |
|  | *GM-CSF* | *2.27  (1.19±2.11)* | *1.00  (-0.00±2.62)* | *0.2349* | *0.1464* |
|  | *gp130/sIL-6Rb* | *38337.55  (15.23±1.11)* | *40174.71  (15.29±1.32)* | *0.9446* | *0.9323* |
|  | *HGF* | *1024.84  (10.00±1.09)* | *563.91  (9.14±1.21)* | *0.1207* | *0.0380* |
|  | *IFN-a2* | *21.08  (4.40±0.85)* | *14.05  (3.81±0.58)* | *0.1017* | *0.0277* |
|  | *IFN-b* | *57.03  (5.83±0.58)* | *48.41  (5.60±0.76)* | *0.4144* | *0.3121* |
|  | *IL-10* | *8.45  (3.08±1.23)* | *4.22  (2.08±1.87)* | *0.1509* | *0.0745* |
|  | *IL-12(p40)* | *209.81  (7.71±0.80)* | *143.01  (7.16±1.03)* | *0.1651* | *0.0908* |
|  | *IL-12(p70)* | *5.41  (2.44±0.58)* | *3.17  (1.66±1.09)* | *0.0595* | *0.0131* |
|  | *IL-15* | *48.31  (5.59±3.34)* | *41.09  (5.36±3.50)* | *0.8223* | *0.7689* |
|  | *IL-16* | *273.03  (8.09±1.14)* | *78.21  (6.29±3.35)* | *0.1207* | *0.0432* |
|  | *IL-17* | *10.86  (3.44±0.68)* | *8.26  (3.05±0.45)* | *0.1430* | *0.0613* |
|  | *IL-18* | *41.68  (5.38±0.73)* | *28.47  (4.83±0.97)* | *0.1651* | *0.0922* |
|  | *IL-19* | *42.09  (5.40±2.00)* | *25.41  (4.67±2.24)* | *0.4160* | *0.3293* |
|  | *IL-1a* | *12.61  (3.66±1.26)* | *5.08  (2.34±1.45)* | *0.0513* | *0.0106* |
|  | *IL-2* | *54.52  (5.77±0.90)* | *34.42  (5.11±1.00)* | *0.1371* | *0.0534* |
|  | *IL-20* | *47.66  (5.57±0.80)* | *29.07  (4.86±0.86)* | *0.0814* | *0.0190* |
|  | *IL-22* | *36.18  (5.18±2.01)* | *16.73  (4.06±1.91)* | *0.1806* | *0.1055* |
|  | *IL-26* | *881.32  (9.78±0.36)* | *769.97  (9.59±0.27)* | *0.1615* | *0.0839* |
|  | *IL-27 (p28)* | *45.98  (5.52±2.09)* | *16.13  (4.01±2.92)* | *0.1676* | *0.0958* |
|  | *IL28A* | *107.15  (6.74±0.60)* | *78.98  (6.30±0.58)* | *0.1207* | *0.0423* |
|  | *IL-29* | *88.83  (6.47±2.37)* | *44.98  (5.49±2.21)* | *0.3229* | *0.2348* |
|  | *IL-2Ra* | *129.63  (7.02±0.52)* | *91.73  (6.52±0.64)* | *0.0932* | *0.0240* |
|  | *IL-32* | *3.34  (1.74±2.51)* | *1.56  (0.65±1.72)* | *0.2393* | *0.1528* |
|  | *IL-34* | *20.23  (4.34±1.55)* | *16.19  (4.02±1.32)* | *0.5687* | *0.4875* |
|  | *IL-35* | *111.79  (6.80±2.96)* | *56.65  (5.82±2.92)* | *0.4160* | *0.3267* |
|  | *IL-4* | *1.05  (0.06±1.99)* | *0.70  (-0.51±1.68)* | *0.4786* | *0.3916* |
|  | *IL-5* | *46.22  (5.53±1.87)* | *13.89  (3.80±3.25)* | *0.1410* | *0.0586* |
|  | *IL-7* | *1.19  (0.25±2.66)* | *1.20  (0.27±2.58)* | *0.9446* | *0.9225* |
|  | *IL-9* | *234.32  (7.87±0.28)* | *199.74  (7.64±0.53)* | *0.2099* | *0.1282* |
|  | *IP-10* | *766.92  (9.58±1.53)* | *363.04  (8.50±1.09)* | *0.1121* | *0.0320* |
|  | *LIGHT/TNFSF14* | *8.60  (3.10±2.65)* | *1.84  (0.88±1.87)* | *0.0513* | *0.0096* |
|  | *MCP-1(MCAF)* | *295.15  (8.21±2.06)* | *131.36  (7.04±1.29)* | *0.1410* | *0.0569* |
|  | *MCP-3* | *2.63  (1.40±2.42)* | *0.70  (-0.51±2.68)* | *0.1207* | *0.0439* |
|  | *MIF* | *7997.28  (12.97±1.17)* | *6991.24  (12.77±1.31)* | *0.6546* | *0.5781* |
|  | *MIG* | *537.24  (9.07±0.54)* | *458.46  (8.84±0.33)* | *0.2393* | *0.1554* |
|  | *MIP-1b* | *181.54  (7.50±0.35)* | *154.70  (7.27±0.41)* | *0.1651* | *0.0883* |
|  | *MMP-1* | *1363.91  (10.41±1.13)* | *1135.67  (10.15±1.44)* | *0.5676* | *0.4792* |
|  | *MMP-2* | *40921.98  (15.32±1.44)* | *41380.59  (15.34±1.33)* | *0.9664* | *0.9664* |
|  | *MMP-3* | *9205.21  (13.17±0.53)* | *6697.16  (12.71±0.77)* | *0.1371* | *0.0531* |
|  | *Osteocalcin* | *3016.84  (11.56±1.08)* | *3542.78  (11.79±1.39)* | *0.6779* | *0.6149* |
|  | *Osteopontin (OPN)* | *55334.14  (15.76±1.26)* | *49187.36  (15.59±1.19)* | *0.6779* | *0.6163* |
|  | *PDGF-bb* | *916.88  (9.84±1.36)* | *859.09  (9.75±1.13)* | *0.8330* | *0.8006* |
|  | *Pentraxin-3* | *37213.27  (15.18±1.52)* | *18212.82  (14.15±1.77)* | *0.1539* | *0.0779* |
|  | *RANTES* | *9522.07  (13.22±1.05)* | *6904.56  (12.75±1.12)* | *0.3229* | *0.2237* |
|  | *sCD163* | *172527.95  (17.40±0.82)* | *156304.52  (17.25±1.35)* | *0.7416* | *0.6838* |
|  | *sCD30/TNFRSF8* | *1020.67  (10.00±0.93)* | *864.67  (9.76±0.90)* | *0.5071* | *0.4215* |
|  | *SCF* | *560.05  (9.13±0.71)* | *469.40  (8.87±0.74)* | *0.3635* | *0.2691* |
|  | *SCGF-b* | *162955.40  (17.31±0.55)* | *128196.74  (16.97±0.47)* | *0.1435* | *0.0652* |
|  | *SDF-1a* | *844.05  (9.72±0.48)* | *1019.75  (9.99±0.36)* | *0.1509* | *0.0735* |
|  | *sIL-6Ra* | *7311.15  (12.84±1.24)* | *5917.07  (12.53±1.55)* | *0.5750* | *0.5003* |
|  | *s-TNF-R1* | *3820.15  (11.90±0.64)* | *2531.89  (11.31±0.85)* | *0.0932* | *0.0242* |
|  | *sTNF-R2* | *815.96  (9.67±0.68)* | *546.98  (9.10±0.85)* | *0.1136* | *0.0339* |
|  | *TNF-b* | *4.52  (2.18±1.87)* | *2.93  (1.55±2.33)* | *0.4160* | *0.3319* |
|  | *TRAIL* | *66.10  (6.05±0.56)* | *57.52  (5.85±0.33)* | *0.3229* | *0.2237* |
|  | *TSLP* | *31.73  (4.99±0.67)* | *21.23*  *(4.41±0.92)* | *0.1207* | *0.0430* |
|  | *TWEAK/TNFSF12* | *151.29  (7.24±0.80)* | *146.83  (7.20±0.95)* | *0.8303* | *0.7871* |
|  | *VEGF* | *230.16  (7.85±0.78)* | *170.15  (7.41±1.36)* | *0.3229* | *0.2327* |
