# Supplementary material for: Expression of S100A Alarmins in Cord Blood Monocytes Is Highly Associated With Chorioamnionitis and Fetal Inflammation in Preterm Infants
Source: Front Immunol. 2020 Jun 16;11:1194. doi: 10.3389/fimmu.2020.01194 (PMC7308505; doi:10.3389/fimmu.2020.01194)
Supplement: Supplementary file 3 [file Table_3.DOCX]

***Supplementary Table 3. Comparison between the results of qPCR analysis and RNA sequencing in samples with high or low S100A expression (n=4 in each group).***

| **gene** | **qPCR** | | | | | **RNA sequencing** | | | | |
| --- | --- | --- | --- | --- | --- | --- | --- | --- | --- | --- |
|  | ***high S100As*** | | ***low S100As*** | |  | ***high S100As*** | | ***low S100As*** | |  |
|  | *average dCt* | *SD* | *average dCt* | *SD* | ***P value*** | *average log2(fpkm)* | *SD* | *average log2(fpkm)* | *SD* | ***P value*** |
| **S100A8** | 5.16 | 0.53 | 2.62 | 0.24 | *0.000* | 15.48 | 0.50 | 12.25 | 0.51 | *0.000* |
| **S100A9** | 5.06 | 0.47 | 3.01 | 0.31 | *0.000* | 15.43 | 0.40 | 12.79 | 0.35 | *< 0.0001* |
| **ANXA3** | -1.41 | 0.87 | -5.49 | 1.08 | *0.001* | 7.79 | 0.90 | 3.38 | 1.34 | *0.002* |
| **FCER1G** | 0.81 | 0.18 | 0.09 | 0.12 | *0.001* | 10.05 | 0.06 | 8.71 | 0.43 | *0.001* |
| **FCGR1A** | -1.18 | 0.56 | -3.17 | 0.21 | *0.001* | 7.12 | 0.67 | 4.12 | 0.70 | *0.001* |
| **LMNB1** | -1.00 | 0.22 | -2.96 | 0.17 | *< 0.0001* | 7.51 | 0.20 | 5.07 | 0.40 | *< 0.0001* |
| **WRN** | -7.22 | 0.21 | -6.28 | 0.30 | *0.002* | 1.20 | 0.26 | 1.71 | 0.35 | *0.059* |

Groups compared by un-paired t test.
