# Supplementary material for: Expression of S100A Alarmins in Cord Blood Monocytes Is Highly Associated With Chorioamnionitis and Fetal Inflammation in Preterm Infants
Source: Front Immunol. 2020 Jun 16;11:1194. doi: 10.3389/fimmu.2020.01194 (PMC7308505; doi:10.3389/fimmu.2020.01194)
Supplement: Supplementary file 4 [file Table_4.DOCX]

***Supplementary Table 4. Fold change and fpkm values for the differentially expressed genes in cord blood monocytes common to high expression of S100A alarmins as well as HCA and FIRS (n=124). The values are given for each group comparison (S100A expression, presence of HCA and FIRS) separately.***

|  | **S100A high/low** | **S100A high** | **S100A low** | **HCA/no HCA** | **HCA** | **no HCA** | **FIRS/no FIRS** | **FIRS** | **no FIRS** |
| --- | --- | --- | --- | --- | --- | --- | --- | --- | --- |
| ***gene symbol*** | ***Fold change*** | ***mean log2(fpkm) (SD)*** | ***mean log2(fpkm) (SD)*** | ***Fold change*** | ***mean log2(fpkm) (SD)*** | ***mean log2(fpkm) (SD)*** | ***Fold change*** | ***mean log2(fpkm) (SD)*** | ***mean log2(fpkm) (SD)*** |
| **ACSS2** | **1,51** | **4,57 (0,33)** | **3,97 (0,35)** | **1,51** | **4,59 (0,32)** | **4 (0,35)** | **1,43** | **4,61 (0,31)** | **4,09 (0,4)** |
| **ACTR3** | **1,48** | **8,33 (0,25)** | **7,76 (0,22)** | **1,36** | **8,29 (0,29)** | **7,85 (0,3)** | **1,27** | **8,28 (0,27)** | **7,93 (0,36)** |
| **ADAM10** | **1,32** | **6,72 (0,22)** | **6,32 (0,22)** | **1,33** | **6,74 (0,23)** | **6,33 (0,2)** | **1,24** | **6,73 (0,24)** | **6,41 (0,27)** |
| **ADCK3** | **0,53** | **2,33 (0,66)** | **3,25 (0,29)** | **0,51** | **2,27 (0,62)** | **3,24 (0,34)** | **0,62** | **2,34 (0,64)** | **3,02 (0,61)** |
| **AKAP1** | **0,51** | **-0,06 (0,46)** | **0,92 (0,45)** | **0,52** | **-0,07 (0,49)** | **0,86 (0,47)** | **0,59** | **-0,08 (0,51)** | **0,69 (0,58)** |
| **ALOX5** | **1,69** | **7,85 (0,29)** | **7,1 (0,22)** | **1,45** | **7,78 (0,4)** | **7,24 (0,36)** | **1,40** | **7,8 (0,4)** | **7,32 (0,4)** |
| **ASGR2** | **0,71** | **-0,94 (1,06)** | **-0,46 (1,17)** | **0,58** | **-1,1 (1,09)** | **-0,33 (1,03)** | **0,68** | **-1,06 (0,92)** | **-0,5 (1,21)** |
| **ATP6V0D1** | **1,54** | **6,56 (0,28)** | **5,94 (0,39)** | **1,55** | **6,59 (0,27)** | **5,96 (0,37)** | **1,40** | **6,57 (0,3)** | **6,09 (0,44)** |
| **B4GALT5** | **2,44** | **5,11 (0,61)** | **3,83 (0,38)** | **2,03** | **5,03 (0,72)** | **4,01 (0,58)** | **1,74** | **5,01 (0,71)** | **4,21 (0,76)** |
| **BATF** | **3,05** | **3,62 (0,65)** | **2,01 (0,63)** | **2,83** | **3,62 (0,65)** | **2,12 (0,76)** | **2,46** | **3,67 (0,6)** | **2,37 (0,93)** |
| **BTAF1** | **0,70** | **4,41 (0,33)** | **4,92 (0,16)** | **0,68** | **4,37 (0,3)** | **4,93 (0,14)** | **0,73** | **4,37 (0,3)** | **4,82 (0,29)** |
| **C17orf62** | **1,61** | **6,52 (0,34)** | **5,83 (0,41)** | **1,53** | **6,51 (0,35)** | **5,89 (0,45)** | **1,40** | **6,5 (0,3)** | **6,01 (0,53)** |
| **C9orf84** | **3,49** | **2,51 (0,77)** | **0,7 (1)** | **2,98** | **2,46 (1)** | **0,89 (0,98)** | **2,37** | **2,44 (1,09)** | **1,2 (1,13)** |
| **CALM3** | **1,64** | **6,42 (0,41)** | **5,71 (0,28)** | **1,65** | **6,45 (0,42)** | **5,73 (0,27)** | **1,54** | **6,47 (0,38)** | **5,85 (0,43)** |
| **CAMK2D** | **0,45** | **2,81 (0,85)** | **3,98 (0,44)** | **0,47** | **2,81 (0,92)** | **3,9 (0,44)** | **0,53** | **2,79 (0,98)** | **3,7 (0,64)** |
| **CARS** | **1,33** | **4,15 (0,2)** | **3,74 (0,24)** | **1,28** | **4,13 (0,26)** | **3,78 (0,22)** | **1,27** | **4,17 (0,29)** | **3,83 (0,22)** |
| **CD177** | **34,29** | **6,22 (1,92)** | **1,12 (2,18)** | **17,50** | **5,94 (2,47)** | **1,81 (2,63)** | **10,60** | **5,97 (2,8)** | **2,57 (2,87)** |
| **CD63** | **2,31** | **8,46 (0,48)** | **7,25 (0,47)** | **2,07** | **8,43 (0,53)** | **7,38 (0,59)** | **1,75** | **8,4 (0,56)** | **7,6 (0,72)** |
| **CEACAM1** | **6,15** | **3,21 (1,35)** | **0,59 (0,58)** | **4,86** | **3,14 (1,48)** | **0,86 (0,99)** | **3,36** | **3,08 (1,57)** | **1,33 (1,43)** |
| **CEACAM3** | **3,07** | **4,7 (0,87)** | **3,08 (1,04)** | **2,98** | **4,73 (0,85)** | **3,16 (1,07)** | **2,37** | **4,71 (0,83)** | **3,47 (1,24)** |
| **CFL1** | **1,62** | **8,97 (0,32)** | **8,27 (0,24)** | **1,52** | **8,95 (0,34)** | **8,35 (0,34)** | **1,36** | **8,92 (0,31)** | **8,48 (0,45)** |
| **CHMP2A** | **1,60** | **5,69 (0,26)** | **5,01 (0,2)** | **1,46** | **5,65 (0,33)** | **5,1 (0,29)** | **1,35** | **5,65 (0,36)** | **5,21 (0,36)** |
| **CNNM3** | **0,64** | **1,12 (0,3)** | **1,76 (0,39)** | **0,66** | **1,12 (0,31)** | **1,72 (0,41)** | **0,70** | **1,11 (0,28)** | **1,61 (0,46)** |
| **CPSF2** | **1,42** | **4,57 (0,26)** | **4,07 (0,25)** | **1,34** | **4,55 (0,28)** | **4,13 (0,3)** | **1,27** | **4,55 (0,18)** | **4,21 (0,38)** |
| **CR1** | **2,91** | **5,89 (0,7)** | **4,35 (0,57)** | **2,61** | **5,87 (0,84)** | **4,49 (0,58)** | **2,22** | **5,88 (0,86)** | **4,73 (0,83)** |
| **CREB5** | **2,03** | **5,94 (0,46)** | **4,92 (0,54)** | **1,96** | **5,96 (0,59)** | **4,99 (0,43)** | **1,70** | **5,94 (0,66)** | **5,18 (0,58)** |
| **CSF2RB** | **2,11** | **6,57 (0,45)** | **5,49 (0,43)** | **1,91** | **6,54 (0,5)** | **5,61 (0,55)** | **1,60** | **6,49 (0,55)** | **5,81 (0,67)** |
| **CYSTM1** | **5,09** | **5,1 (0,82)** | **2,75 (0,63)** | **3,66** | **4,96 (1,1)** | **3,09 (0,97)** | **2,62** | **4,88 (1,12)** | **3,49 (1,3)** |
| **DOK3** | **1,82** | **5,74 (0,36)** | **4,88 (0,42)** | **1,79** | **5,77 (0,36)** | **4,93 (0,44)** | **1,56** | **5,74 (0,38)** | **5,1 (0,56)** |
| **DYRK2** | **0,71** | **2,05 (0,34)** | **2,55 (0,23)** | **0,67** | **1,99 (0,27)** | **2,57 (0,23)** | **0,76** | **2,04 (0,26)** | **2,43 (0,38)** |
| **EIF2AK4** | **0,69** | **2,62 (0,3)** | **3,15 (0,17)** | **0,70** | **2,61 (0,32)** | **3,13 (0,15)** | **0,77** | **2,63 (0,34)** | **3,01 (0,29)** |
| **ERI1** | **1,76** | **2,91 (0,54)** | **2,09 (0,39)** | **1,65** | **2,89 (0,58)** | **2,17 (0,43)** | **1,53** | **2,91 (0,59)** | **2,29 (0,53)** |
| **EXOC6** | **1,98** | **5,06 (0,33)** | **4,07 (0,21)** | **1,62** | **4,95 (0,47)** | **4,26 (0,44)** | **1,49** | **4,96 (0,48)** | **4,39 (0,52)** |
| **EXOC7** | **1,43** | **5,01 (0,3)** | **4,49 (0,14)** | **1,41** | **5,02 (0,28)** | **4,52 (0,21)** | **1,37** | **5,05 (0,3)** | **4,59 (0,26)** |
| **FAM109A** | **0,58** | **0,38 (0,38)** | **1,17 (0,19)** | **0,62** | **0,4 (0,47)** | **1,09 (0,22)** | **0,72** | **0,44 (0,5)** | **0,93 (0,41)** |
| **FAM151B** | **1,84** | **3,11 (0,48)** | **2,23 (0,43)** | **1,73** | **3,1 (0,56)** | **2,31 (0,42)** | **1,62** | **3,13 (0,58)** | **2,44 (0,51)** |
| **FAM20A** | **1,29** | **0 (0,96)** | **-0,37 (0,92)** | **1,27** | **0 (0,98)** | **-0,34 (0,9)** | **1,25** | **0,02 (1,08)** | **-0,29 (0,84)** |
| **FCER1G** | **1,87** | **9,61 (0,41)** | **8,71 (0,37)** | **1,85** | **9,64 (0,42)** | **8,75 (0,36)** | **1,61** | **9,62 (0,45)** | **8,94 (0,52)** |
| **FCGR1A** | **4,20** | **6,23 (0,89)** | **4,16 (0,99)** | **3,72** | **6,22 (0,95)** | **4,33 (1,1)** | **2,98** | **6,24 (0,94)** | **4,67 (1,3)** |
| **FCGR1B** | **3,43** | **4,24 (0,76)** | **2,46 (0,85)** | **2,91** | **4,2 (0,85)** | **2,65 (0,99)** | **2,60** | **4,27 (0,87)** | **2,89 (1,07)** |
| **FCGR1C** | **3,69** | **2,21 (0,86)** | **0,33 (0,97)** | **2,89** | **2,11 (0,99)** | **0,58 (1,15)** | **2,76** | **2,24 (0,87)** | **0,78 (1,23)** |
| **FKBP1A** | **1,49** | **7,16 (0,24)** | **6,59 (0,31)** | **1,39** | **7,14 (0,26)** | **6,66 (0,37)** | **1,35** | **7,17 (0,26)** | **6,73 (0,38)** |
| **FNDC3B** | **1,64** | **6,46 (0,33)** | **5,74 (0,28)** | **1,56** | **6,45 (0,38)** | **5,8 (0,3)** | **1,47** | **6,47 (0,43)** | **5,91 (0,36)** |
| **FXN** | **0,38** | **0,75 (0,89)** | **2,14 (0,54)** | **0,39** | **0,71 (0,96)** | **2,07 (0,47)** | **0,49** | **0,76 (1,04)** | **1,78 (0,79)** |
| **GBA** | **1,95** | **4,1 (0,7)** | **3,13 (0,31)** | **2,02** | **4,15 (0,71)** | **3,14 (0,23)** | **1,86** | **4,2 (0,75)** | **3,3 (0,47)** |
| **GFRA2** | **0,20** | **-0,33 (1,18)** | **2 (0,88)** | **0,24** | **-0,3 (1,28)** | **1,79 (1,06)** | **0,35** | **-0,19 (1,4)** | **1,33 (1,4)** |
| **GK** | **2,95** | **5,66 (0,65)** | **4,11 (0,95)** | **2,78** | **5,68 (0,71)** | **4,21 (0,95)** | **2,25** | **5,66 (0,8)** | **4,5 (1,06)** |
| **GNG5** | **1,55** | **7,05 (0,32)** | **6,42 (0,24)** | **1,45** | **7,02 (0,34)** | **6,49 (0,32)** | **1,33** | **7,01 (0,36)** | **6,6 (0,39)** |
| **GPR84** | **3,28** | **3,83 (0,98)** | **2,11 (0,97)** | **3,18** | **3,87 (0,95)** | **2,2 (1,03)** | **2,65** | **3,9 (0,98)** | **2,49 (1,18)** |
| **GTF2A2** | **1,53** | **5,49 (0,3)** | **4,88 (0,41)** | **1,46** | **5,48 (0,37)** | **4,94 (0,4)** | **1,37** | **5,49 (0,41)** | **5,04 (0,42)** |
| **GUSBP3** | **2,21** | **3,24 (0,45)** | **2,1 (0,61)** | **2,00** | **3,21 (0,6)** | **2,21 (0,6)** | **1,78** | **3,22 (0,67)** | **2,39 (0,66)** |
| **HCK** | **1,59** | **8,02 (0,26)** | **7,35 (0,33)** | **1,48** | **7,99 (0,26)** | **7,43 (0,42)** | **1,37** | **7,99 (0,25)** | **7,54 (0,46)** |
| **HDAC1** | **1,32** | **5,38 (0,21)** | **4,98 (0,24)** | **1,31** | **5,39 (0,23)** | **5 (0,22)** | **1,24** | **5,38 (0,24)** | **5,07 (0,27)** |
| **HSD3B7** | **4,47** | **0,4 (1,11)** | **-1,76 (0,92)** | **3,96** | **0,4 (1,28)** | **-1,59 (0,91)** | **3,19** | **0,44 (1,33)** | **-1,24 (1,21)** |
| **IFNAR1** | **1,61** | **4,92 (0,28)** | **4,23 (0,3)** | **1,51** | **4,9 (0,33)** | **4,31 (0,35)** | **1,40** | **4,9 (0,36)** | **4,42 (0,4)** |
| **IGSF6** | **1,65** | **7,03 (0,37)** | **6,31 (0,39)** | **1,57** | **7,02 (0,4)** | **6,37 (0,42)** | **1,42** | **7 (0,44)** | **6,5 (0,48)** |
| **IL4R** | **2,80** | **6,03 (0,51)** | **4,54 (0,42)** | **2,36** | **5,96 (0,61)** | **4,72 (0,65)** | **1,96** | **5,94 (0,65)** | **4,97 (0,81)** |
| **ITGB2** | **1,49** | **8,9 (0,32)** | **8,33 (0,18)** | **1,51** | **8,93 (0,3)** | **8,34 (0,18)** | **1,41** | **8,94 (0,3)** | **8,45 (0,31)** |
| **JAK3** | **2,22** | **5,13 (0,48)** | **3,97 (0,72)** | **2,17** | **5,15 (0,49)** | **4,04 (0,73)** | **1,93** | **5,17 (0,54)** | **4,23 (0,78)** |
| **KDM1A** | **0,78** | **3,31 (0,19)** | **3,67 (0,16)** | **0,80** | **3,31 (0,22)** | **3,64 (0,16)** | **0,82** | **3,3 (0,24)** | **3,59 (0,18)** |
| **KREMEN1** | **6,39** | **2,42 (0,98)** | **-0,26 (1,03)** | **4,96** | **2,34 (1,23)** | **0,03 (1,21)** | **3,35** | **2,27 (1,33)** | **0,52 (1,54)** |
| **L3MBTL3** | **0,66** | **2,61 (0,42)** | **3,2 (0,2)** | **0,66** | **2,58 (0,41)** | **3,18 (0,24)** | **0,72** | **2,59 (0,43)** | **3,07 (0,36)** |
| **LAMTOR2** | **1,49** | **5,14 (0,3)** | **4,56 (0,36)** | **1,42** | **5,13 (0,33)** | **4,62 (0,38)** | **1,35** | **5,14 (0,35)** | **4,7 (0,4)** |
| **LCP1** | **1,49** | **9,49 (0,23)** | **8,92 (0,2)** | **1,40** | **9,47 (0,26)** | **8,98 (0,28)** | **1,29** | **9,46 (0,26)** | **9,08 (0,35)** |
| **LGALS1** | **1,98** | **9,96 (0,44)** | **8,98 (0,48)** | **1,78** | **9,93 (0,5)** | **9,09 (0,57)** | **1,55** | **9,9 (0,41)** | **9,26 (0,7)** |
| **LIMK2** | **3,69** | **5,77 (0,65)** | **3,89 (0,67)** | **2,79** | **5,65 (0,87)** | **4,16 (0,9)** | **2,23** | **5,62 (0,99)** | **4,46 (1,03)** |
| **LITAF** | **2,61** | **7,32 (0,65)** | **5,94 (0,5)** | **2,35** | **7,3 (0,71)** | **6,07 (0,62)** | **2,02** | **7,31 (0,79)** | **6,29 (0,75)** |
| **LMNB1** | **3,43** | **6,86 (0,66)** | **5,08 (0,66)** | **2,74** | **6,77 (0,85)** | **5,32 (0,83)** | **2,15** | **6,73 (0,88)** | **5,62 (1,04)** |
| **LUC7L** | **0,73** | **4,33 (0,24)** | **4,8 (0,16)** | **0,75** | **4,33 (0,28)** | **4,76 (0,16)** | **0,78** | **4,33 (0,3)** | **4,68 (0,23)** |
| **MAP3K14-AS1** | **0,51** | **-0,47 (0,6)** | **0,51 (0,55)** | **0,54** | **-0,46 (0,65)** | **0,42 (0,58)** | **0,60** | **-0,47 (0,74)** | **0,26 (0,63)** |
| **MAP4K1** | **0,43** | **2,18 (0,9)** | **3,4 (0,41)** | **0,41** | **2,1 (0,89)** | **3,39 (0,38)** | **0,44** | **2,01 (0,89)** | **3,21 (0,63)** |
| **MARVELD1** | **0,32** | **0,32 (0,96)** | **1,96 (0,74)** | **0,29** | **0,2 (0,83)** | **1,97 (0,75)** | **0,41** | **0,28 (0,89)** | **1,58 (1,09)** |
| **METTL7B** | **15,99** | **0,83 (2,33)** | **-3,16 (1,82)** | **15,42** | **0,96 (2,48)** | **-2,99 (1,68)** | **11,35** | **1,13 (2,42)** | **-2,37 (2,31)** |
| **MILR1** | **1,82** | **4,96 (0,43)** | **4,09 (0,46)** | **1,64** | **4,92 (0,49)** | **4,2 (0,52)** | **1,62** | **4,98 (0,5)** | **4,29 (0,53)** |
| **MRPL28** | **1,40** | **4,31 (0,2)** | **3,82 (0,3)** | **1,33** | **4,3 (0,27)** | **3,88 (0,3)** | **1,26** | **4,29 (0,27)** | **3,96 (0,34)** |
| **MTR** | **0,72** | **2,4 (0,19)** | **2,87 (0,19)** | **0,76** | **2,42 (0,22)** | **2,82 (0,23)** | **0,79** | **2,41 (0,25)** | **2,75 (0,26)** |
| **MTRR** | **1,89** | **4,46 (0,53)** | **3,54 (0,28)** | **1,98** | **4,53 (0,48)** | **3,54 (0,26)** | **1,63** | **4,47 (0,41)** | **3,76 (0,61)** |
| **MYO10** | **3,49** | **2,2 (0,91)** | **0,39 (0,91)** | **3,87** | **2,33 (0,8)** | **0,38 (0,84)** | **3,00** | **2,33 (0,85)** | **0,75 (1,12)** |
| **MYO7B** | **3,27** | **0,34 (0,92)** | **-1,37 (1,02)** | **3,37** | **0,42 (0,89)** | **-1,33 (0,99)** | **2,99** | **0,52 (0,96)** | **-1,06 (1,08)** |
| **NCF4** | **2,25** | **6,97 (0,37)** | **5,8 (0,37)** | **1,89** | **6,89 (0,48)** | **5,97 (0,56)** | **1,59** | **6,84 (0,48)** | **6,18 (0,7)** |
| **PART1** | **0,32** | **-1,13 (1,16)** | **0,49 (0,64)** | **0,35** | **-1,14 (1,19)** | **0,37 (0,78)** | **0,47** | **-1,06 (0,81)** | **0,04 (1,33)** |
| **PIK3AP1** | **1,86** | **6,96 (0,47)** | **6,07 (0,47)** | **1,85** | **6,99 (0,48)** | **6,1 (0,46)** | **1,70** | **7,02 (0,45)** | **6,25 (0,58)** |
| **PIK3IP1** | **3,23** | **1,74 (0,72)** | **0,05 (0,82)** | **2,67** | **1,67 (0,9)** | **0,25 (0,89)** | **2,13** | **1,63 (1,01)** | **0,54 (1,03)** |
| **PIM1** | **2,53** | **6,33 (0,68)** | **5 (0,85)** | **2,38** | **6,34 (0,68)** | **5,09 (0,9)** | **1,99** | **6,33 (0,63)** | **5,33 (1,03)** |
| **PLB1** | **1,75** | **4,91 (0,41)** | **4,11 (0,43)** | **1,63** | **4,89 (0,42)** | **4,19 (0,5)** | **1,52** | **4,91 (0,46)** | **4,31 (0,53)** |
| **PLSCR1** | **2,35** | **8,06 (0,49)** | **6,83 (0,85)** | **2,26** | **8,08 (0,46)** | **6,9 (0,89)** | **1,85** | **8,04 (0,5)** | **7,15 (0,96)** |
| **PLXNC1** | **1,52** | **6,25 (0,29)** | **5,65 (0,34)** | **1,45** | **6,25 (0,4)** | **5,71 (0,27)** | **1,37** | **6,26 (0,45)** | **5,8 (0,32)** |
| **POLR1E** | **0,59** | **0,5 (0,45)** | **1,26 (0,48)** | **0,61** | **0,49 (0,52)** | **1,2 (0,44)** | **0,67** | **0,5 (0,53)** | **1,07 (0,53)** |
| **PPM1M** | **1,62** | **5,48 (0,31)** | **4,78 (0,2)** | **1,46** | **5,43 (0,36)** | **4,88 (0,33)** | **1,36** | **5,43 (0,35)** | **4,99 (0,41)** |
| **PPP1R18** | **1,41** | **5,77 (0,21)** | **5,28 (0,18)** | **1,33** | **5,75 (0,25)** | **5,34 (0,23)** | **1,26** | **5,75 (0,26)** | **5,42 (0,28)** |
| **PSMB7** | **0,99** | **2,02 (0,84)** | **2,03 (0,77)** | **1,20** | **2,16 (0,83)** | **1,89 (0,77)** | **1,27** | **2,23 (0,91)** | **1,89 (0,7)** |
| **PTPN2** | **1,43** | **5,78 (0,27)** | **5,27 (0,25)** | **1,34** | **5,76 (0,31)** | **5,34 (0,29)** | **1,29** | **5,77 (0,31)** | **5,41 (0,33)** |
| **RAB31** | **1,51** | **6,32 (0,36)** | **5,72 (0,29)** | **1,42** | **6,29 (0,43)** | **5,79 (0,3)** | **1,34** | **6,3 (0,46)** | **5,88 (0,35)** |
| **RGL4** | **4,77** | **5,59 (0,88)** | **3,33 (0,64)** | **3,39** | **5,43 (1,1)** | **3,67 (1,03)** | **2,50** | **5,37 (1,07)** | **4,05 (1,33)** |
| **RHBDD2** | **1,74** | **3,34 (0,54)** | **2,55 (0,33)** | **1,71** | **3,36 (0,54)** | **2,59 (0,36)** | **1,58** | **3,38 (0,59)** | **2,72 (0,46)** |
| **RHOG** | **1,61** | **6,54 (0,26)** | **5,86 (0,18)** | **1,52** | **6,53 (0,28)** | **5,92 (0,29)** | **1,43** | **6,54 (0,28)** | **6,03 (0,36)** |
| **S100A11** | **1,66** | **1,03 (0,93)** | **0,3 (0,94)** | **1,30** | **0,89 (0,98)** | **0,51 (1)** | **1,13** | **0,81 (0,96)** | **0,63 (1,03)** |
| **SBNO2** | **2,33** | **4,98 (0,48)** | **3,77 (0,65)** | **2,00** | **4,93 (0,5)** | **3,92 (0,8)** | **1,74** | **4,92 (0,56)** | **4,12 (0,84)** |
| **SERPINA1** | **1,47** | **0,1 (1,73)** | **-0,46 (1,08)** | **0,89** | **-0,23 (1,65)** | **-0,07 (1,33)** | **0,77** | **-0,38 (1,79)** | **0,01 (1,26)** |
| **SIPA1L2** | **2,28** | **3,79 (0,69)** | **2,6 (0,54)** | **2,17** | **3,8 (0,75)** | **2,69 (0,55)** | **1,77** | **3,75 (0,82)** | **2,93 (0,74)** |
| **SLC2A3** | **2,97** | **2,92 (0,72)** | **1,35 (0,78)** | **2,28** | **9,14 (0,62)** | **7,96 (0,91)** | **1,93** | **9,13 (0,68)** | **8,19 (0,97)** |
| **SNX20** | **1,48** | **4,82 (0,35)** | **4,26 (0,33)** | **1,50** | **4,85 (0,31)** | **4,27 (0,36)** | **1,41** | **4,86 (0,31)** | **4,37 (0,41)** |
| **SRF** | **1,44** | **3,39 (0,33)** | **2,87 (0,25)** | **1,45** | **3,41 (0,3)** | **2,88 (0,28)** | **1,34** | **3,41 (0,34)** | **2,98 (0,34)** |
| **SRP14** | **1,48** | **6,69 (0,24)** | **6,13 (0,19)** | **1,42** | **6,68 (0,27)** | **6,18 (0,23)** | **1,31** | **6,67 (0,3)** | **6,28 (0,3)** |
| **ST3GAL2** | **1,55** | **3,91 (0,31)** | **3,28 (0,35)** | **1,51** | **3,92 (0,31)** | **3,32 (0,37)** | **1,43** | **3,94 (0,26)** | **3,42 (0,44)** |
| **TCAIM** | **1,35** | **3,7 (0,3)** | **3,27 (0,46)** | **1,36** | **3,73 (0,31)** | **3,28 (0,43)** | **1,30** | **3,74 (0,32)** | **3,36 (0,44)** |
| **TESC** | **1,69** | **5,1 (0,45)** | **4,34 (0,45)** | **1,73** | **5,14 (0,42)** | **4,35 (0,45)** | **1,51** | **5,11 (0,34)** | **4,52 (0,6)** |
| **TMBIM6** | **1,39** | **7,66 (0,29)** | **7,18 (0,2)** | **1,33** | **7,65 (0,31)** | **7,23 (0,24)** | **1,26** | **7,64 (0,33)** | **7,31 (0,3)** |
| **TMEM117** | **2,48** | **0,84 (0,71)** | **-0,47 (0,81)** | **2,58** | **0,91 (0,58)** | **-0,45 (0,85)** | **2,29** | **0,96 (0,65)** | **-0,23 (0,9)** |
| **TMEM120A** | **1,62** | **4,8 (0,36)** | **4,1 (0,24)** | **1,51** | **4,78 (0,39)** | **4,18 (0,34)** | **1,37** | **4,76 (0,39)** | **4,3 (0,44)** |
| **TNFRSF1A** | **1,49** | **6,33 (0,3)** | **5,76 (0,32)** | **1,40** | **6,31 (0,34)** | **5,83 (0,35)** | **1,35** | **6,33 (0,38)** | **5,9 (0,36)** |
| **TPRKBP2** | **2,84** | **2,63 (0,78)** | **1,12 (0,64)** | **2,69** | **2,65 (0,81)** | **1,22 (0,69)** | **2,16** | **2,62 (0,86)** | **1,51 (0,92)** |
| **TYSND1** | **0,51** | **-0,13 (0,66)** | **0,85 (0,33)** | **0,54** | **-0,11 (0,73)** | **0,77 (0,37)** | **0,62** | **-0,11 (0,8)** | **0,59 (0,52)** |
| **UBE2L3** | **1,37** | **4,6 (0,33)** | **4,14 (0,17)** | **1,33** | **4,6 (0,35)** | **4,19 (0,19)** | **1,27** | **4,6 (0,33)** | **4,26 (0,3)** |
| **UFD1L** | **1,46** | **4,62 (0,29)** | **4,08 (0,23)** | **1,45** | **4,64 (0,29)** | **4,1 (0,23)** | **1,30** | **4,61 (0,25)** | **4,23 (0,37)** |
| **URB1** | **0,51** | **-0,26 (0,33)** | **0,72 (0,52)** | **0,55** | **-0,24 (0,4)** | **0,63 (0,55)** | **0,64** | **-0,2 (0,43)** | **0,44 (0,65)** |
| **WDFY3** | **1,69** | **4,51 (0,31)** | **3,75 (0,26)** | **1,51** | **4,46 (0,39)** | **3,86 (0,35)** | **1,39** | **4,45 (0,41)** | **3,98 (0,43)** |
| **WDFY3-AS1** | **1,80** | **6,12 (0,37)** | **5,27 (0,45)** | **1,70** | **6,11 (0,49)** | **5,35 (0,4)** | **1,49** | **6,09 (0,55)** | **5,51 (0,5)** |
| **WDR59** | **1,76** | **3,09 (0,43)** | **2,28 (0,53)** | **1,71** | **3,1 (0,49)** | **2,32 (0,48)** | **1,56** | **3,11 (0,52)** | **2,46 (0,55)** |
| **VOPP1** | **0,76** | **2,69 (0,17)** | **3,08 (0,15)** | **0,79** | **2,7 (0,21)** | **3,04 (0,17)** | **0,82** | **2,69 (0,23)** | **2,98 (0,2)** |
| **ZBTB41** | **0,70** | **1,53 (0,36)** | **2,05 (0,22)** | **0,68** | **1,49 (0,34)** | **2,05 (0,21)** | **0,73** | **1,49 (0,38)** | **1,94 (0,31)** |
| **ZNF337** | **0,62** | **1,32 (0,44)** | **2,02 (0,22)** | **0,61** | **1,29 (0,44)** | **2 (0,23)** | **0,68** | **1,3 (0,4)** | **1,85 (0,44)** |
| **ZNF438** | **1,82** | **3,14 (0,43)** | **2,28 (0,38)** | **1,67** | **3,11 (0,55)** | **2,37 (0,35)** | **1,55** | **3,13 (0,61)** | **2,5 (0,44)** |
| **ZNF529** | **0,68** | **1,24 (0,26)** | **1,8 (0,38)** | **0,68** | **1,22 (0,26)** | **1,78 (0,37)** | **0,74** | **1,23 (0,28)** | **1,67 (0,42)** |
