# Supplementary material for: Expression of S100A Alarmins in Cord Blood Monocytes Is Highly Associated With Chorioamnionitis and Fetal Inflammation in Preterm Infants
Source: Front Immunol. 2020 Jun 16;11:1194. doi: 10.3389/fimmu.2020.01194 (PMC7308505; doi:10.3389/fimmu.2020.01194)
Supplement: Supplementary file 5 [file Table_5.DOCX]

***Supplementary Table 5. Hub genes detected in the yellow module presented in order of connectivity.*** *13 DE genes common to high expression of S100A alarmins, fetal inflammatory response syndrome (FIRS) and histological chorioamnionitis (HCA) are presented in red*

| **Gene name** | **Full name,**  **short description** | **Within module connectivity** |
| --- | --- | --- |
| ANXA3 | *annexin A3,*  a member of calcium-dependent phospholipid-binding protein family, plays a role in the inhibition of phopholipase A2 and cleavage of inositol 1,2-cyclic phosphate to form inositol 1-phosphate. | 97.85 |
| **CYSTM1** | *cysteine rich transmembrane module containing 1*,  - | 96.30 |
| BASP1 | *brain abundant membrane attached signal protein 1,*  membrane bound protein with several transient phosphorylation sites and PEST motifs | 93.11 |
| **NCF4** | *neutrophil cytosolic factor 4*,  a cytosolic regulatory component of the superoxide-producing phagocyte NADPH-oxidase | 91.70 |
| **IL4R** | *interleukin 4 receptor*,  encodes the alpha chain of the interleukin-4 receptor, a type I transmembrane protein that can bind interleukin 4 and interleukin 13 | 90.14 |
| **RGL4** | *ral guanine nucleotide dissociation stimulator like 4*,  a protein similar to guanine nucleotide exchange factor Ral guanine dissociation stimulator | 88.93 |
| **B4GALT5** | *beta-1,4-galactosyltransferase 5*,  type II membrane-bound glycoproteins that appear to have exclusive specificity for the donor substrate UDPgalactose | 87.71 |
| PPP1R3B | *protein phosphatase 1 regulatory subunit 3B,*  catalytic subunit of the serine/theonine phosphatase, protein phosphatase-1, may be involved in regulating glycogen synthesis. | 86.37 |
| GCA | *Grancalcin,*  calcium-binding protein that is abundant in neutrophils and macrophages. Depending on divalent cations, this protein can be localized in cytosolic or granule fraction | 83.46 |
| FAM129A | *niban apoptosis regulator 1,*  member of the family with sequence similarity 129 protein family, may play a role in regulating p53-mediated apoptosis | 82.74 |
| **LMNB1** | *lamin B1*,  B-type lamin protein, is a component of the nuclear lamina | 82.35 |
| UBAP1 | *ubiquitin associated protein 1,*  member of the UBA domain family connected to ubiquitin and the ubiquitination pathway | 81.28 |
| FPR2 | *formyl peptide receptor 2,*  - | 81.27 |
| STAT3 | *signal transducer and activator of transcription 3,*  member of the STAT protein family. In response to cytokines and growth factors, STAT family members translocate to the cell nucleus where they act as transcription activators, plays a key role in many cellular processes such as cell growth and apoptosis. | 80.92 |
| GPR97 | *adhesion G protein-coupled receptor G3,*  - | 79.49 |
| DGAT2 | *diacylglycerol O-acyltransferase 2,*  enzyme which catalyzes the final reaction in the synthesis of triglycerides in which diacylglycerol is covalently bound to long chain fatty acyl-CoAs at low concentrations of magnesium chloride | 78.80 |
| S100A8 | *S100 calcium binding protein A8,*  member of the S100 family of proteins containing 2 EF-hand calcium-binding motifs. Involved in the regulation of a number of cellular processes such as cell cycle progression and differentiation, may function in the inhibition of casein kinase and as a cytokine. | 78.39 |
| **LIMK2** | *LIM domain kinase 2*,  belongs to a small subfamily of LIM proteins with 2 Nterminal LIM motifs and a C-terminal protein kinase domain, phosphorylates cofilin, inhibiting its actindepolymerizing activity | 77.70 |
| S100A9 | *S100 calcium binding protein A9,*  member of the S100 family of proteins containing 2 EF-hand calcium-binding motifs. Iinvolved in the regulation of a number of cellular processes such as cell cycle progression and differentiation, may function in the inhibition of casein kinase, may exhibit antifungal and antibacterial activity | 77.26 |
| FLOT1 | *flotillin 1,*  protein that localizes to the caveolae, plays a role in vesicle trafficking and cell morphology | 76.62 |
| GYG1 | *glycogenin 1,*  member of the glycogenin family, involved in glycogen synthesis. This protein is a glycosyltransferase that catalyzes the formation of a short glucose polymer from uridine diphosphate glucose in an autoglucosylation reaction. | 75.43 |
| FPR1 | *formyl peptide receptor 1,*  a G protein-coupled receptor of mammalian phagocytic cells, mediates the response of phagocytic cells to invasion of the host by microorganisms and is important in host defense and inflammation | 75.07 |
| PROK2 | *prokineticin 2,*  a protein expressed in the suprachiasmatic nucleus (SCN) circadian clock that may function as the output component of the circadian clock. The secreted form of the encoded protein may also serve as a chemoattractant for neuronal precursor cells in the olfactory bulb | 74.81 |
| PYGL | *glycogen phosphorylase L,*  a homodimeric protein that catalyses the cleavage of alpha-1,4-glucosidic bonds to release glucose-1-phosphate from liver glycogen stores | 74.40 |
| DCPS | *decapping enzyme, scavenger,*  a member of the histidine triad family of pyrophosphatases that removes short mRNA fragments which appear in the mRNA decay pathway, following deadenylation and exosome-mediated turnover. It protects the cell from the potentially toxic accumulation of these short, capped mRNA fragments, and regulates the activity of other cap-binding proteins, which are inhibited by their accumulation. It also acts as a transcript-specific modulator of pre-mRNA splicing and microRNA turnover | 74.10 |
| PGS1 | *phosphatidylglycerophosphate synthase 1,*  - | 73.36 |
| **KREMEN1** | *kringle containing transmembrane protein 1*,  a high-affinity dickkopf homolog 1 (DKK1) transmembrane receptor that functionally cooperates with DKK1 to block wingless (WNT)/beta-catenin signaling | 70.58 |
| STAT5B | *signal transducer and activator of transcription 5B,*  member of the STAT protein family. In response to cytokines and growth factors, STAT family members translocate to the cell nucleus where they act as transcription activators, plays a key role in many cellular processes such as TCR signaling, apoptosis, adult mammary gland development, and sexual dimorphism of liver gene expression. | 69.39 |
| GLT1D1 | *glycosyltransferase 1 domain containing 1,*  - | 68.68 |
| TGFA | *transforming growth factor alpha,*  a growth factor, a ligand for the epidermal growth factor receptor, which activates a signaling pathway for cell proliferation, differentiation and development. This protein may act as either a transmembrane-bound ligand or a soluble ligand. | 68.39 |
| ALPL | *alkaline phosphatase, biomineralization associated,*  a member of the alkaline phosphatase family of proteins, a membrane bound glycosylated enzyme that is referred to as the tissue-nonspecific form of the enzyme, may play a role in bone mineralization | 68.31 |
| **CD177** | *CD177 molecule*,  a glycosyl-phosphatidylinositol (GPI)-linked cell surface glycoprotein that plays a role in neutrophil activation | 68.31 |
| **LITAF** | *lipopolysaccharide induced TNF factor*,  lipopolysaccharide-induced TNF-alpha factor, which is a DNA-binding protein and can mediate the TNF-alpha expression by direct binding to the promoter region of the TNF-alpha gene | 68.13 |
| AQP9 | *aquaporin 9,*  belongs to a family of water-selective membrane channels. This gene encodes a member of a subset of aquaporins called the aquaglyceroporins. This protein allows passage of a broad range of noncharged solutes and also stimulates urea transport and osmotic water permeability. This protein may also facilitate the uptake of glycerol in hepatic tissue and may also play a role in specialized leukocyte functions such as immunological response and bactericidal activity | 67.64 |
| HSDL2 | *hydroxysteroid dehydrogenase like 2,*  - | 67.05 |
| SH3GLB1 | *SH3 domain containing GRB2 like, endophilin B1,*  a SRC homology 3 domain-containing protein, interacts with the proapoptotic member of the Bcl-2 family, Bcl-2-associated X protein (Bax) and may be involved in regulating apoptotic signaling pathways, may also be involved in maintaining mitochondrial morphology | 66.91 |
| **MYO10** | *myosin X*,  a member of the myosin superfamily, represents an unconventional myosin | 66.51 |
| **CSF2RB** | *colony stimulating factor 2 receptor beta common subunit*,  common beta chain of the high affinity receptor for IL-3, IL-5 and CSF | 66.32 |
| ANKRD22 | *ankyrin repeat domain 22,*  - | 65.47 |
| **EXOC6** | *exocyst complex component 6*,  similar to the yeast gene product, which is essential for vesicular traffic from the Golgi apparatus to the cell surface, one of the components of a multiprotein complex required for exocytosis | 64.56 |
| CST7 | *cystatin F,*  belongs to cystatin superfamily. Some of the members are active cysteine protease inhibitors, while others have lost or perhaps never acquired this inhibitory activity. This gene encodes a glycosylated cysteine protease inhibitor with a putative role in immune regulation through inhibition of a unique target in the hematopoietic system | 63.97 |

List of hub genes was created using R package WGCNA available from its web site (https://horvath.genetics.ucla.edu/html/CoexpressionNetwork/Rpackages/WGCNA/), gene official full name, info about corresponding protein transcript are adapted from Gene ncbi database https://www.ncbi.nlm.nih.gov/gene/
