# Supplementary material for: Expression of S100A Alarmins in Cord Blood Monocytes Is Highly Associated With Chorioamnionitis and Fetal Inflammation in Preterm Infants
Source: Front Immunol. 2020 Jun 16;11:1194. doi: 10.3389/fimmu.2020.01194 (PMC7308505; doi:10.3389/fimmu.2020.01194)
Supplement: Supplementary file 6 [file Table_6.DOCX]

***Supplementary Table 6. Results of Gene Ontology Enrichment analysis for all genes within the yellow module as identified by network analysis.*** *Terms clearly related to inflammation and immunity are marked in red.*

| **Term Name** | **Term ID** | **Term Definition** | **Bonferoni corrected p-value for the enrichment score** |
| --- | --- | --- | --- |
| inflammatory response | GO:0006954 | The immediate defensive reaction (by vertebrate tissue) to infection or injury caused by chemical or physical agents. The process is characterized by local vasodilation, extravasation of plasma into intercellular spaces and accumulation of white blood cells and macrophages. | 2,23E-07 |
| myeloid leukocyte activation | GO:0002274 | A change in the morphology or behavior of a myeloid leukocyte resulting from exposure to an activating factor such as a cellular or soluble ligand. | 1,07E-05 |
| plasma membrane | GO:0005886 | The membrane surrounding a cell that separates the cell from its external environment. It consists of a phospholipid bilayer and associated proteins. | 2,96E-05 |
| cell periphery | GO:0071944 | The part of a cell encompassing the cell cortex, the plasma membrane, and any external encapsulating structures. | 5,62E-05 |
| specific granule | GO:0042581 | Granule with a membranous, tubular internal structure, found primarily in mature neutrophil cells. Most are released into the extracellular fluid. Specific granules contain lactoferrin, lysozyme, vitamin B12 binding protein and elastase. | 0,0001 |
| leukocyte activation | GO:0045321 | A change in morphology and behavior of a leukocyte resulting from exposure to a specific antigen, mitogen, cytokine, cellular ligand, or soluble factor. | 0,0007 |
| defense response | GO:0006952 | Reactions, triggered in response to the presence of a foreign body or the occurrence of an injury, which result in restriction of damage to the organism attacked or prevention/recovery from the infection caused by the attack. | 0,0008 |
| leukocyte degranulation | GO:0043299 | The regulated exocytosis of secretory granules by a leukocyte. | 0,0022 |
| cell activation | GO:0001775 | A change in the morphology or behavior of a cell resulting from exposure to an activating factor such as a cellular or soluble ligand. | 0,0029 |
| specific granule lumen | GO:0035580 | The volume enclosed by the membrane of a specific granule, a granule with a membranous, tubular internal structure, found primarily in mature neutrophil cells. Most are released into the extracellular fluid. Specific granules contain lactoferrin, lysozyme, vitamin B12 binding protein and elastase. | 0,0050 |
