# Supplementary figures and images for: Expression of S100A Alarmins in Cord Blood Monocytes Is Highly Associated With Chorioamnionitis and Fetal Inflammation in Preterm Infants
Source: Front Immunol. 2020 Jun 16;11:1194. doi: 10.3389/fimmu.2020.01194 (PMC7308505; doi:10.3389/fimmu.2020.01194)

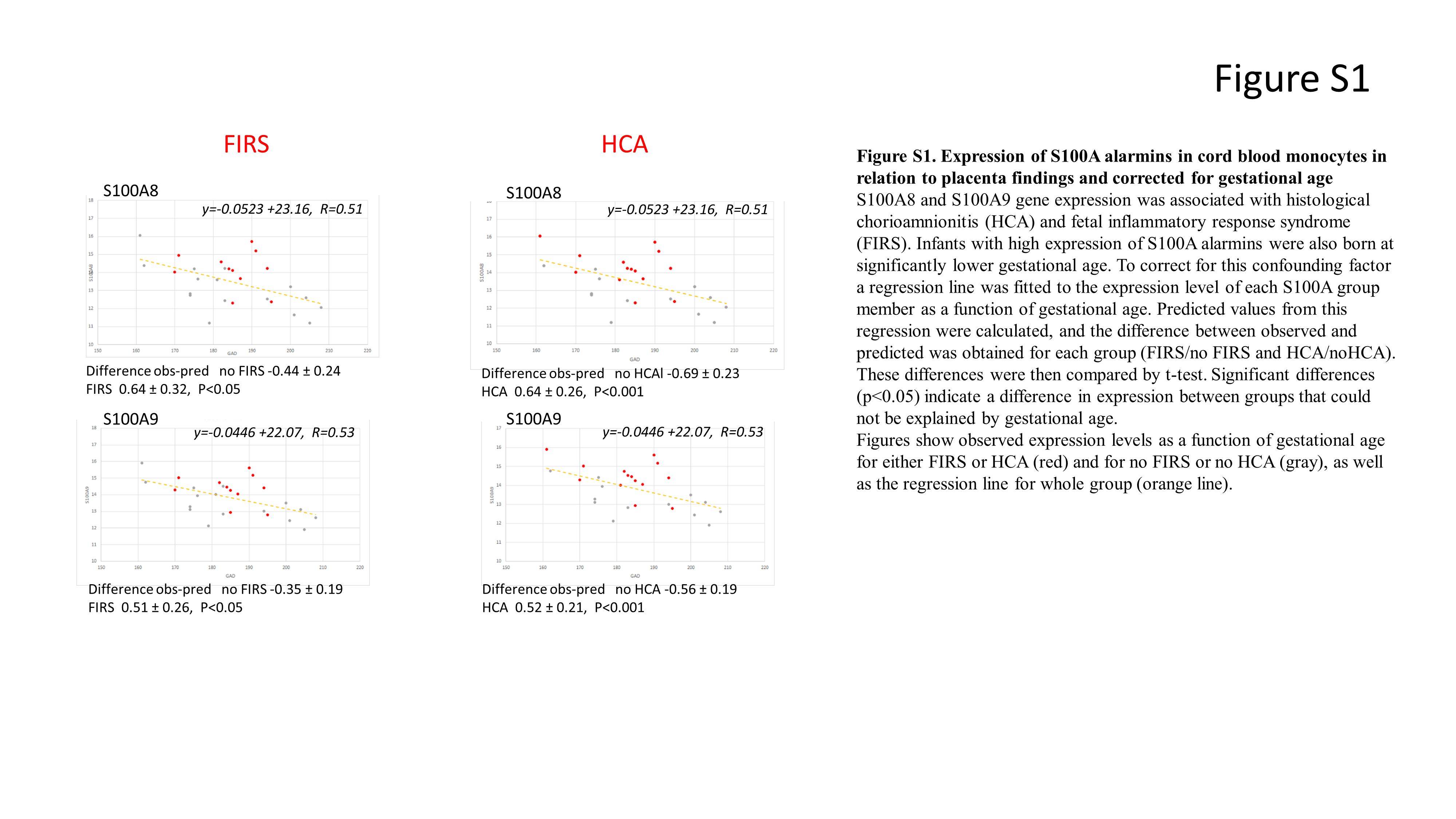

Supplement: Supplementary file 7 [file Image_1.TIF]
